# Supplementary material for: Defining and measuring multimorbidity in primary care in Singapore: Results of an online Delphi study
Source: PLoS One. 2022 Dec 1;17(12):e0278559. doi: 10.1371/journal.pone.0278559 (PMC9714819; doi:10.1371/journal.pone.0278559)
Supplement: S2 Appendix — (DOCX) [file pone.0278559.s002.docx]

**S2 Appendix**. Delphi Round 1 Survey

This first round of Delphi will ask you about issues related to the operationalisation of multimorbidity and selection of chronic conditions to classify patients as multi-morbid. The purpose is for generation of ideas and identification of salient issues to guide subsequent Delphi rounds. Please respond to the questions below in the text box provided. Please be as detailed in your response as possible. (e.g., if it is applicable, describe a condition or scenario to better articulate a point).

# Section A [Defining and Operationalising Multimorbidity]

1. How would you define a chronic condition?
   1. *Probe: factors to consider like duration of disease, curable or not, trajectory of disease, impact on patient/healthcare system.*

|  |
| --- |

1. Please describe what you understand by the term multimorbidity?

|  |
| --- |

1. Are you aware of any definitions of multimorbidity that you came across in literature?
   1. Yes -> **Go to Q4**
   2. No -> **Go to Q6**
2. Please elaborate or share the citation for the definition(s)

|  |
| --- |

1. Do you feel the above definition(s) are comprehensive enough to describe multimorbidity? If not, please share what is lacking or can be improved?

|  |
| --- |

1. Do you think that using ‘counts of chronic conditions’ is sufficient to identify patients with multimorbidity? Please elaborate.

|  |
| --- |

1. In your opinion, how many chronic conditions (i.e., cut-off) should be used to identify patients with multimorbidity?

|  |
| --- |

1. What factors should be considered when choosing the cut-off to identify patients with multimorbidity?
   1. *Probe: some considerations could be to better differentiate more or less patients as multi-morbid, to better differentiate only those patients with a higher disease burden, dependent on purpose of defining multimorbidity like for research or management of patients etc.*

|  |
| --- |

1. In your opinion, how important is ‘chronic pain’ to be considered as part of the list of conditions to define multimorbidity? Please elaborate.

|  |
| --- |

1. How should we measure the burden of multimorbidity?
   1. *Probe: from system’s perspective, provider’s perspective, from patient’s perspective*

|  |
| --- |

1. In your opinion, what types of data should be used for obtaining information of individuals’ chronic disease(s) to measure multimorbidity in the general population. Please elaborate.
   1. *Probe: administrative records, self-reported, insurance claims etc.*

|  |
| --- |

# Section B [Proposed List of Chronic Conditions]

With a list of chronic conditions for studying multimorbidity in primary care as proposed by international researchers (1), multimorbidity researchers within Singapore developed a context-specific and locally relevant list of chronic conditions for studying multimorbidity in primary care in Singapore. Please refer below:

| **S/N** | **Conditions** | **ICD-10 Codes** |
| --- | --- | --- |
| 1 | Hyperlipidaemia | E78.5 (Hyperlipidaemia, unspecified) |
| 2 | Hypertension (high blood pressure) | I10 (Essential (primary) hypertension) |
| 3 | Diabetes  (including pre-diabetes) | E09 (Impaired glucose regulation) |
|  |  | E099 (Impaired glucose regulation without complication) |
|  |  | E10.9 (Type 1 diabetes mellitus without complication) |
|  |  | E11.9 (Type 2 diabetes mellitus without complication) |
|  |  | E14.2 (Diabetes mellitus with incipient diabetic nephropathy) |
|  |  | E14.3 (Diabetes mellitus with retinopathy) |
|  |  | E14.31 (Unspecified diabetes mellitus with background retinopathy) |
|  |  | E14.64 (Unspecified diabetes mellitus with hypoglycaemia) |
|  |  | E14.73 (Unspecified diabetes mellitus with foot ulcer due to multiple causes) |
| 4 | Arthritis &/or rheumatoid arthritis | M06.99 (Rheumatoid arthritis, unspecified, site unspecified) |
|  |  | M15.9 (Osteoarthritis (OA) - Generalised) |
|  |  | M19.99 (Arthritis, unspecified, site unspecified) |
| 5 | Obesity | E66.9 (Obesity, unspecified) |
| 6 | Cardiovascular disease (angina, MI, AF, poor circulation of lower limbs) | I25.9 (Chronic ischaemic heart disease, unspecified) |
|  |  | I48 (Atrial fibrillation and flutter) |
|  |  | I70.20 (Atherosclerosis of arteries of extremities, unspecified) |
|  |  | I73.9 (Peripheral vascular disease, unspecified) |
| 7 | Asthma, COPD, or chronic bronchitis | J44.9 (Chronic Obstructive Pulmonary Disease, Unspecified) |
|  |  | J45.9 (Asthma, unspecified) |
| 8 | Chronic hepatitis | K76.9 (Liver disease, unspecified) |
|  |  | Z22.51 (Carrier of viral hepatitis B) |
| 9 | Stomach problem (reflux, heartburn, or gastric ulcer) | K21.9 (Gastro-oesophageal reflux disease without oesophagitis) |
|  |  | K27.9 (Peptic ulcer, unspecified as acute or chronic, without haemorrhage or perforation) |
| 10 | Thyroid disorder | E03.9 (Hypothyroidism, unspecified) |
|  |  | E05.9 (Thyrotoxicosis, unspecified) |
| 11 | Stroke and TIA | G45.9 (Transient cerebral ischaemic attack, unspecified) |
|  |  | I64 (Stroke, not specified as haemorrhage or infarction) |
| 12 | Heart failure (including valve problems or replacement) | I50.0 (Congestive heart failure) |
|  |  | I51.9 (Heart disease, unspecified) |
| 13 | Kidney disease or failure | N03.9 (Unspecified nephritic syndrome, unspecified) |
|  |  | N18.9 (Chronic kidney disease, unspecified) |
| 14 | Depression or anxiety | F32.20 (Severe depressive episode without psychotic symptoms, not specified as arising in the postnatal period) |
|  |  | F32.90 (Depressive episode, unspecified, not specified as arising in the postnatal period) |
|  |  | F41.1 (Anxiety disorder, unspecified) |
| 15 | Chronic urinary problem | N40 (Hyperplasia of prostate) |
| 16 | Physical disability | H91.9 (Hearing loss, unspecified) |
|  |  | Q79.9 (Congenital malformation of musculoskeletal system, unspecified) |
| 17 | Any cancer in the last 5 years | C80 (Malignant neoplasm without specification of site) |
| 18 | Osteoporosis | M81.99 (Other osteoporosis, site unspecified) |
| 19 | Dementia or Alzheimer's disease | F03 (Unspecified dementia) |
| 20 | Colon problem (irritable bowel) | K58.9 (Irritable bowel syndrome without diarrhoea) |

We would like to seek your feedback on this list.

1. Please share your views on the appropriateness of the list of chronic conditions presented here.
   1. *Probe: some conditions listed may not be suitable in Singapore healthcare context, some appropriate conditions may be missing from this list.*

|  |
| --- |

1. In your clinical practice, do you have any challenges in coding the list of chronic conditions presented here?
   1. *Yes* -> **Go to Q14**
   2. *No* -> **Go to Q15**
2. Could you elaborate/describe the challenges?
   1. *Probe: IT support, human factors like time constraints, medical coding system etc.*

|  |
| --- |

1. Please share your views on other mental conditions (apart from anxiety and depression) which should be included in the list. Please provide some examples.
   1. *Probe: Schizophrenia, Bipolar Disorder etc.*

|  |
| --- |

1. Please share your views on other physical disability conditions (apart from hearing loss and congenital malformation of musculoskeletal system) which should be included in the list. Please provide some examples.
   1. *Probe – amputations, blindness etc.*

|  |
| --- |

1. In your opinion, should socio-demographic characteristics (e.g., deprivation index etc.) be part of the list of conditions to define multimorbidity? Please elaborate.

|  |
| --- |

1. Please provide any other feedback on the list presented.

|  |
| --- |

Thank you for your participation, we look forward to your continuous participation in subsequent Delphi rounds.

**References**

1. Fortin M, Almirall J, Nicholson K. Development of a research tool to document self-reported chronic conditions in primary care. J Comorb. 2017;7(1):117-123. Published 2017 Nov 9. doi:10.15256/joc.2017.7.122
