# Supplementary material for: Defining and measuring multimorbidity in primary care in Singapore: Results of an online Delphi study
Source: PLoS One. 2022 Dec 1;17(12):e0278559. doi: 10.1371/journal.pone.0278559 (PMC9714819; doi:10.1371/journal.pone.0278559)
Supplement: S3 Appendix — (DOCX) [file pone.0278559.s003.docx]

**S3 Appendix**. Delphi Round 2 Survey

Thank you for providing your valuable feedback to the questions in Delphi Round 1. Based on the findings of Delphi Round 1 (which we have shared with you in the Round 2 study invite email), we have developed Round 2 survey questions. Most of the options for questions below are developed from the responses received in Round 1 from the panellists. Definitions for certain terms are provided to facilitate answering of questions. We invite you to answer these questions to facilitate developing a common agreed upon definition of multimorbidity in Singapore. We strongly recommend that you read the summary of findings and multimorbidity (brief information) pdf slides before attempting Round 2 questions.

# Section A (Defining and Operationalizing Multimorbidity)

1. **Please enter your assigned Participant Number here**: ____________

Since majority of the panellists indicated the need for a more comprehensive definition of multimorbidity using chronic conditions, we will define multimorbidity based on the following 5 constructs:

1. the definition of chronic conditions,
2. number of conditions in the multimorbidity list,
3. cut-off for number of conditions to define multimorbidity,
4. data source(s) to confirm conditions and
5. severity of included conditions.

Please provide your feedback to the questions below related to these 5 constructs of the multimorbidity definition.

1. [a] Definition of Chronic Condition:

**Select all that are needed to define a chronic condition from the list below**:

- 1. Duration of condition
  2. Impact on patient
  3. Incurable condition
  4. Management of patient (e.g., long-term follow-up)
  5. Recurrent or persistent course of condition
  6. Sequelae of condition

1. **For a condition to be defined as chronic, the condition should last for:**
   1. 1 month or more
   2. 3 months or more
   3. 6 months or more
   4. 12 months or more
2. **For a condition to be defined as chronic, the impact on patient should be measured as: (select all that apply)**
   1. Limitations in activities of daily living or instrumental activities of daily living or physical disability
   2. Mortality
   3. Psychological impairment
   4. Social Deprivation (defined as limited access to society's resources due to poverty, discrimination, or other disadvantage).
3. [b] Number of Conditions in the Multimorbidity List: Based on panellists’ responses in Round 1, the list of conditions is expanded to include 27 conditions (from original 20 conditions). Please ***click here*** to access the expanded list of 27 conditions.

**Do you agree to define multimorbidity with this expanded list of 27 conditions (which fulfils the minimum criteria of at least 12 conditions suggested by Fortin et al [1]) in the Singapore context?**

(Reference 1: Fortin M, Almirall J, Nicholson K. Development of a research tool to document self-reported chronic conditions in primary care. J Comorb. 2017;7(1):117-123. Published 2017 Nov 9. doi:10.15256/joc.2017.7.122)

- 1. Yes
  2. No

1. [c] Cut-off for Number of Conditions to Define Multimorbidity: Majority of the panellists in Round 1 indicated 3 or more conditions as the cut-off for defining multimorbidity.

**Do you agree with this cut-off of 3 or more conditions?**

- 1. Yes
  2. No

[d] Data Source(s) to Confirm Conditions: Panellists shared in Round 1 that multimorbidity should be measured from 3 perspectives: patient, provider and health system. Please indicate the most appropriate data source(s) for confirming multimorbidity from each perspective. (*Note: Ministry of Health administrative data is defined as a comprehensive database maintained by the Ministry of Health in Singapore which comprises of the following: health services utilization data, claims data and patient medical records*).

1. **Most appropriate data source(s) for measuring multimorbidity from patients’ perspective (e.g., quality of life, self-rated health, treatment burden etc.) is/are**:
   1. Ministry of Health Administrative Data
   2. Electronic Medical Records
   3. Medications or Pharmacy Data
   4. Patient Self-Reported Outcomes (collected via patient interviews, focussed group discussions, surveys etc.)
   5. Data on Social Determinants (e.g., MSW reports, caregiver information etc.)
2. **Most appropriate data source(s) for measuring multimorbidity from providers’ perspective (e.g., safety, consult time etc.) is/are**:
   1. Ministry of Health Administrative Data
   2. Electronic Medical Records
   3. Medications or Pharmacy Data
   4. Patient Self-Reported Outcomes (collected via patient interviews, focussed group discussions, surveys etc.)
   5. Data on Social Determinants (e.g., MSW reports, caregiver information etc.)
3. **Most appropriate data source(s) for measuring multimorbidity from health system’s perspective is/are:**
   1. Ministry of Health Administrative Data
   2. Electronic Medical Records
   3. Medications or Pharmacy Data
   4. Patient Self-Reported Outcomes (collected via patient interviews, focussed group discussions, surveys etc.)
   5. Data on Social Determinants (e.g., MSW reports, caregiver information etc.)
4. [e] Severity of Included Conditions: Severity of chronic conditions is an important factor to consider when defining the *impact* of multimorbidity on outcomes like treatment burden, resource use and associated cost, quality of life etc. However, for estimation of prevalence of multimorbidity at national level (e.g., to track longitudinally), counts of chronic conditions should suffice.

**Do you agree with this statement?**

- 1. Yes
  2. No

1. **Please provide additional comments or suggestions related to the above question 10, if any, in the space below**.

|  |
| --- |

# Section B [Proposed Expanded List of Chronic Conditions]

With a list of chronic conditions for studying multimorbidity in primary care as proposed by international researchers [1], multimorbidity researchers within Singapore developed a context-specific and locally relevant list of chronic conditions for studying multimorbidity in primary care in Singapore, which was shared with panellists in Delphi Round 1. Based on recommendations from Round 1, this list is expanded to include 27 conditions.

(Reference 1: Fortin M, Almirall J, Nicholson K. Development of a research tool to document self-reported chronic conditions in primary care. J Comorb. 2017;7(1):117-123. Published 2017 Nov 9. doi:10.15256/joc.2017.7.122)

Please ***click here*** to access the expanded list of 27 conditions:

| **Expanded List of Conditions (Additions are Indicated in Blue)** | | |
| --- | --- | --- |
| **S/N** | **Conditions** | **ICD-10 Codes** |
| 1 | Hyperlipidaemia | E78.5 (Hyperlipidaemia, unspecified) |
| 2 | Hypertension (high blood pressure) | I10 (Essential (primary) hypertension) |
| 3 | Diabetes  (including pre-diabetes) | E09 (Impaired glucose regulation) |
|  |  | E099 (Impaired glucose regulation without complication) |
|  |  | E10.9 (Type 1 diabetes mellitus without complication) |
|  |  | E11.9 (Type 2 diabetes mellitus without complication) |
|  |  | E14.2 (Diabetes mellitus with incipient diabetic nephropathy) |
|  |  | E14.3 (Diabetes mellitus with retinopathy) |
|  |  | E14.31 (Unspecified diabetes mellitus with background retinopathy) |
|  |  | E14.64 (Unspecified diabetes mellitus with hypoglycaemia) |
|  |  | E14.73 (Unspecified diabetes mellitus with foot ulcer due to multiple causes) |
| 4 | Arthritis &/or rheumatoid arthritis | M06.99 (Rheumatoid arthritis, unspecified, site unspecified) |
|  |  | M15.9 (Osteoarthritis (OA) - Generalised) |
|  |  | M19.99 (Arthritis, unspecified, site unspecified) |
| 5 | Obesity | E66.9 (Obesity, unspecified) |
| 6 | Cardiovascular disease (angina, MI, AF, poor circulation of lower limbs) | I25.9 (Chronic ischaemic heart disease, unspecified) |
|  |  | I48 (Atrial fibrillation and flutter) |
|  |  | I70.20 (Atherosclerosis of arteries of extremities, unspecified) |
|  |  | I73.9 (Peripheral vascular disease, unspecified) |
| 7 | Asthma, COPD, or chronic bronchitis | J44.9 (Chronic Obstructive Pulmonary Disease, Unspecified) |
|  |  | J45.9 (Asthma, unspecified) |
| 8 | Chronic hepatitis | K76.9 (Liver disease, unspecified) |
|  |  | Z22.51 (Carrier of viral hepatitis B) |
| 9 | Stomach problem (reflux, heartburn, or gastric ulcer) | K21.9 (Gastro-oesophageal reflux disease without oesophagitis) |
|  |  | K27.9 (Peptic ulcer, unspecified as acute or chronic, without haemorrhage or perforation) |
| 10 | Thyroid disorder | E03.9 (Hypothyroidism, unspecified) |
|  |  | E05.9 (Thyrotoxicosis, unspecified) |
| 11 | Stroke and TIA | G45.9 (Transient cerebral ischaemic attack, unspecified) |
|  |  | I64 (Stroke, not specified as haemorrhage or infarction) |
| 12 | Heart failure (including valve problems or replacement) | I50.0 (Congestive heart failure) |
|  |  | I51.9 (Heart disease, unspecified) |
| 13 | Kidney disease or failure | N03.9 (Unspecified nephritic syndrome, unspecified) |
|  |  | N18.9 (Chronic kidney disease, unspecified) |
| 14 | Depression or anxiety | F32.20 (Severe depressive episode without psychotic symptoms, not specified as arising in the postnatal period) |
|  |  | F32.90 (Depressive episode, unspecified, not specified as arising in the postnatal period) |
|  |  | F41.1 (Anxiety disorder, unspecified) |
| 15 | Chronic urinary problem | N40 (Hyperplasia of prostate), |
|  |  | N39 (Other disorders of urinary system) |
|  |  | N20.9 (Urinary calculus, unspecified) |
|  |  | Incontinence* |
| 16 | Functional Limitation | H91.9 (Hearing loss, unspecified) |
|  |  | Q79.9 (Congenital malformation of musculoskeletal system, unspecified) |
|  |  | H26.9 (Cataract, unspecified) |
|  |  | H54.9 (Unspecified visual impairment) |
|  |  | Q89.9 (Congenital malformation, unspecified) |
|  |  | Z89.4 (Acquired absence of foot and ankle) |
|  |  | Z89.5 (Acquired absence of leg at or below knee) |
|  |  | Z89.6 (Acquired absence of leg above knee) |
|  |  | M67.99 (Disorder of synovium and tendon, unspecified) |
|  |  | M79.89 (Other specified soft tissue disorders, site unspecified) |
|  |  | Paraplegia* |
|  |  | Hemiplegia* |
|  |  | Enthesopathy* |
| 17 | Cognitive Limitation | G80.9 (Cerebral palsy, unspecified) |
|  |  | Q90.9 (Down's syndrome, unspecified) |
|  |  | F79.9 (Unspecified mental retardation without mention of impairment of behaviour) |
|  |  | Autism* |
|  |  | ADHD* |
| 18 | Any cancer in the last 5 years | C80 (Malignant neoplasm without specification of site) |
| 19 | Osteoporosis | M81.99 (Other osteoporosis, site unspecified) |
| 20 | Dementia or Alzheimer's disease | F03 (Unspecified dementia) |
| 21 | Colon problem (irritable bowel) | K58.9 (Irritable bowel syndrome without diarrhoea) |
| 22 | Skin Conditions | L70.9 (Other Acne) |
|  |  | L20.8 (Other Atopic Dermatitis) |
|  |  | L40.0 (Psoriasis Vulgaris) |
|  |  | L40.8 (Other Psoriasis) |
| 23 | Chronic Pain | Pain* |
|  |  | Chronic Fatigue* |
|  |  | Fibromyalgia* |
| 24 | Allergic rhinitis | J30.4 (Allergic rhinitis, unspecified) |
| 25 | Gout | M10.9 (Gout, unspecified) |
|  |  | M10.99 (Gout, unspecified, site unspecified) |
| 26 | Other Mental Health Conditions | F20.9 (Schizophrenia, unspecified) |
|  |  | F22.9 (Delusional disorder) |
|  |  | F29 (Unspecified nonorganic psychosis) |
|  |  | F31.9 (Bipolar affective disorder, unspecified) |
|  |  | F48.9 (Neurotic disorder) |
|  |  | F55.9 (Unspecified harmful use of non-dependence producing substance) |
|  |  | F99 (Mental disorder, not otherwise specified) |
|  |  | G47.0 (Disorders of initiating and maintaining sleep [insomnias]) |
|  |  | Z86.5 (Personal history of other mental and behavioural disorders) |
|  |  | PTSD* |
|  |  | OCD* |
|  |  | Chronic narcotic dependency syndrome/drug abuse* |
|  |  | Personality disorder* |
|  |  | Phobia* |
|  |  | Somatoform disorders/somatic symptom disorder* |
|  |  | Eating disorders* |
|  |  | Alcohol abuse* |
|  |  | Burnout* |
| 27 | Neurological Disorders | G40.90 Epilepsy, unspecified, without mention of intractable epilepsy |
|  |  | G20 Parkinson's disease |
|  | *: ICD-10 codes are not used in Polyclinic Coding | |

No panellist suggested to remove any condition from the original list of 20 conditions shared in Delphi Round 1, hence we have retained all these conditions to get feedback in Round 2.

**For subsequent questions, please rate each of the chronic conditions in the order of importance (1: limited importance and exclude to 9: critical importance and include) to be included in the final list of chronic conditions to be used for studying multimorbidity in primary care in Singapore.**

1. **Hyperlipidaemia with following ICD-10 coding: (1) E78.5 (Hyperlipidaemia, unspecified)**

| Limited Importance |  |  |  |  |  |  |  | Critical Importance |
| --- | --- | --- | --- | --- | --- | --- | --- | --- |
| 1 | 2 | 3 | 4 | 5 | 6 | 7 | 8 | 9 |
|  |  |  |  |  |  |  |  |  |
|  |  |  |  |  |  |  |  |  |

1. **Please provide additional comments or suggestions, if any:**

|  |
| --- |

1. **Hypertension (high blood pressure) with following ICD-10 coding: (1) I10 (Essential (primary) hypertension)**

| Limited Importance |  |  |  |  |  |  |  | Critical Importance |
| --- | --- | --- | --- | --- | --- | --- | --- | --- |
| 1 | 2 | 3 | 4 | 5 | 6 | 7 | 8 | 9 |
|  |  |  |  |  |  |  |  |  |
|  |  |  |  |  |  |  |  |  |

1. **Please provide additional comments or suggestions, if any:**

|  |
| --- |

1. **Diabetes (including pre-diabetes) with following ICD-10 codes: (1) E09 (Impaired glucose regulation), (2) E099 (Impaired glucose regulation without complication), (3) E10.9 (Type 1 diabetes mellitus without complication), (4) E11.9 (Type 2 diabetes mellitus without complication), (5) E14.2 (Diabetes mellitus with incipient diabetic nephropathy), (6) E14.3 (Diabetes mellitus with retinopathy), (7) E14.31 (Unspecified diabetes mellitus with background retinopathy), (8) E14.64 (unspecified diabetes mellitus with hypoglycaemia), (9) E14.73 (Unspecified diabetes mellitus with foot ulcer due to multiple causes)**

| Limited Importance |  |  |  |  |  |  |  | Critical Importance |
| --- | --- | --- | --- | --- | --- | --- | --- | --- |
| 1 | 2 | 3 | 4 | 5 | 6 | 7 | 8 | 9 |
|  |  |  |  |  |  |  |  |  |
|  |  |  |  |  |  |  |  |  |

1. **Please provide additional comments or suggestions, if any:**

|  |
| --- |

1. **Arthritis and/or rheumatoid arthritis with following ICD-10 codes: (1) M06.99 (Rheumatoid arthritis, unspecified, site unspecified), (2) M15.9 (Osteoarthritis (OA) - Generalised), (3) M19.99 (Arthritis, unspecified, site unspecified)**

| Limited Importance |  |  |  |  |  |  |  | Critical Importance |
| --- | --- | --- | --- | --- | --- | --- | --- | --- |
| 1 | 2 | 3 | 4 | 5 | 6 | 7 | 8 | 9 |
|  |  |  |  |  |  |  |  |  |
|  |  |  |  |  |  |  |  |  |

1. **Please provide additional comments or suggestions, if any:**

|  |
| --- |

1. **Obesity with following ICD codes: (1) E66.9 (Obesity, unspecified)**

| Limited Importance |  |  |  |  |  |  |  | Critical Importance |
| --- | --- | --- | --- | --- | --- | --- | --- | --- |
| 1 | 2 | 3 | 4 | 5 | 6 | 7 | 8 | 9 |
|  |  |  |  |  |  |  |  |  |
|  |  |  |  |  |  |  |  |  |

1. **Please provide additional comments or suggestions, if any:**

|  |
| --- |

1. **Cardiovascular disease (angina, MI, AF, poor circulation of lower limbs) with following ICD-10 codes: (1) I25.9 (Chronic ischaemic heart disease, unspecified), (2) I48 (Atrial fibrillation and flutter), (3) I70.20 (Atherosclerosis of arteries of extremities, unspecified), (4) I73.9 (Peripheral vascular disease, unspecified)**

| Limited Importance |  |  |  |  |  |  |  | Critical Importance |
| --- | --- | --- | --- | --- | --- | --- | --- | --- |
| 1 | 2 | 3 | 4 | 5 | 6 | 7 | 8 | 9 |
|  |  |  |  |  |  |  |  |  |
|  |  |  |  |  |  |  |  |  |

1. **Please provide additional comments or suggestions, if any:**

|  |
| --- |

1. **Asthma, COPD, or chronic bronchitis with following ICD-10 codes: (1) J44.9 (Chronic Obstructive Pulmonary Disease, Unspecified), (2) J45.9 (Asthma, unspecified)**

| Limited Importance |  |  |  |  |  |  |  | Critical Importance |
| --- | --- | --- | --- | --- | --- | --- | --- | --- |
| 1 | 2 | 3 | 4 | 5 | 6 | 7 | 8 | 9 |
|  |  |  |  |  |  |  |  |  |
|  |  |  |  |  |  |  |  |  |

1. **Please provide additional comments or suggestions, if any:**

|  |
| --- |

1. **Chronic hepatitis with following ICD-10 codes: (1) K76.9 (Liver disease, unspecified), (2) Z22.51 (Carrier of viral hepatitis B)**

| Limited Importance |  |  |  |  |  |  |  | Critical Importance |
| --- | --- | --- | --- | --- | --- | --- | --- | --- |
| 1 | 2 | 3 | 4 | 5 | 6 | 7 | 8 | 9 |
|  |  |  |  |  |  |  |  |  |
|  |  |  |  |  |  |  |  |  |

1. **Please provide additional comments or suggestions, if any:**

|  |
| --- |

1. **Stomach problem (reflux, heartburn, or gastric ulcer) with following ICD codes: (1) K21.9 (Gastro-oesophageal reflux disease without oesophagitis), (2) K27.9 (Peptic ulcer, unspecified as acute or chronic, without haemorrhage or perforation)**

| Limited Importance |  |  |  |  |  |  |  | Critical Importance |
| --- | --- | --- | --- | --- | --- | --- | --- | --- |
| 1 | 2 | 3 | 4 | 5 | 6 | 7 | 8 | 9 |
|  |  |  |  |  |  |  |  |  |
|  |  |  |  |  |  |  |  |  |

1. **Please provide additional comments or suggestions, if any:**

|  |
| --- |

1. **Thyroid disorder with following ICD-10 codes: (1) E03.9 (Hypothyroidism, unspecified), (2) E05.9 (Thyrotoxicosis, unspecified)**

| Limited Importance |  |  |  |  |  |  |  | Critical Importance |
| --- | --- | --- | --- | --- | --- | --- | --- | --- |
| 1 | 2 | 3 | 4 | 5 | 6 | 7 | 8 | 9 |
|  |  |  |  |  |  |  |  |  |
|  |  |  |  |  |  |  |  |  |

1. **Please provide additional comments or suggestions, if any:**

|  |
| --- |

1. **Stroke and TIA with following ICD-10 codes: (1) G45.9 (Transient cerebral ischaemic attack, unspecified), (2) I64 (Stroke, not specified as haemorrhage or infarction)**

| Limited Importance |  |  |  |  |  |  |  | Critical Importance |
| --- | --- | --- | --- | --- | --- | --- | --- | --- |
| 1 | 2 | 3 | 4 | 5 | 6 | 7 | 8 | 9 |
|  |  |  |  |  |  |  |  |  |
|  |  |  |  |  |  |  |  |  |

1. **Please provide additional comments or suggestions, if any:**

|  |
| --- |

1. **Heart failure (including valve problems or replacement) with following ICD-10 codes: (1) I50.0 (Congestive heart failure), (2) I51.9 (Heart disease, unspecified)**

| Limited Importance |  |  |  |  |  |  |  | Critical Importance |
| --- | --- | --- | --- | --- | --- | --- | --- | --- |
| 1 | 2 | 3 | 4 | 5 | 6 | 7 | 8 | 9 |
|  |  |  |  |  |  |  |  |  |
|  |  |  |  |  |  |  |  |  |

1. **Please provide additional comments or suggestions, if any:**

|  |
| --- |

1. **Kidney disease or failure with following ICD-10 codes: (1) N03.9 (Unspecified nephritic syndrome, unspecified), (2) N18.9 (Chronic kidney disease, unspecified)**

| Limited Importance |  |  |  |  |  |  |  | Critical Importance |
| --- | --- | --- | --- | --- | --- | --- | --- | --- |
| 1 | 2 | 3 | 4 | 5 | 6 | 7 | 8 | 9 |
|  |  |  |  |  |  |  |  |  |
|  |  |  |  |  |  |  |  |  |

1. **Please provide additional comments or suggestions, if any:**

|  |
| --- |

1. **Depression or anxiety with following ICD-10 codes: (1) F32.20 (Severe depressive episode without psychotic symptoms, not specified as arising in the postnatal period), (2) F32.90 (Depressive episode, unspecified, not specified as arising in the postnatal period), (3) F41.1 (Anxiety disorder, unspecified)**

| Limited Importance |  |  |  |  |  |  |  | Critical Importance |
| --- | --- | --- | --- | --- | --- | --- | --- | --- |
| 1 | 2 | 3 | 4 | 5 | 6 | 7 | 8 | 9 |
|  |  |  |  |  |  |  |  |  |
|  |  |  |  |  |  |  |  |  |

1. **Please provide additional comments or suggestions, if any:**

|  |
| --- |

1. **Chronic urinary problem with following ICD-10 codes: (1) N40 (Hyperplasia of prostate), N39 (Other disorders of urinary system), N20.9 (Urinary calculus, unspecified) and Incontinence*** (*: ICD-10 codes are not used in Polyclinic Coding)

| Limited Importance |  |  |  |  |  |  |  | Critical Importance |
| --- | --- | --- | --- | --- | --- | --- | --- | --- |
| 1 | 2 | 3 | 4 | 5 | 6 | 7 | 8 | 9 |
|  |  |  |  |  |  |  |  |  |
|  |  |  |  |  |  |  |  |  |

1. **Please provide additional comments or suggestions, if any:**

|  |
| --- |

1. **Any cancer in the last 5 years with following ICD-10 codes: (1) C80 (Malignant neoplasm without specification of site)**

| Limited Importance |  |  |  |  |  |  |  | Critical Importance |
| --- | --- | --- | --- | --- | --- | --- | --- | --- |
| 1 | 2 | 3 | 4 | 5 | 6 | 7 | 8 | 9 |
|  |  |  |  |  |  |  |  |  |
|  |  |  |  |  |  |  |  |  |

1. **Please provide additional comments or suggestions, if any:**

|  |
| --- |

1. **Osteoporosis with following ICD-10 codes: (1) M81.99 (Other osteoporosis, site unspecified)**

| Limited Importance |  |  |  |  |  |  |  | Critical Importance |
| --- | --- | --- | --- | --- | --- | --- | --- | --- |
| 1 | 2 | 3 | 4 | 5 | 6 | 7 | 8 | 9 |
|  |  |  |  |  |  |  |  |  |
|  |  |  |  |  |  |  |  |  |

1. **Please provide additional comments or suggestions, if any:**

|  |
| --- |

1. **Dementia or Alzheimer’s disease with following ICD-10 codes: (1) F03 (Unspecified dementia)**

| Limited Importance |  |  |  |  |  |  |  | Critical Importance |
| --- | --- | --- | --- | --- | --- | --- | --- | --- |
| 1 | 2 | 3 | 4 | 5 | 6 | 7 | 8 | 9 |
|  |  |  |  |  |  |  |  |  |
|  |  |  |  |  |  |  |  |  |

1. **Please provide additional comments or suggestions, if any:**

|  |
| --- |

1. **Colon problem (irritable bowel) with following ICD-10 codes: (1) K58.9 (Irritable bowel syndrome without diarrhoea)**

| Limited Importance |  |  |  |  |  |  |  | Critical Importance |
| --- | --- | --- | --- | --- | --- | --- | --- | --- |
| 1 | 2 | 3 | 4 | 5 | 6 | 7 | 8 | 9 |
|  |  |  |  |  |  |  |  |  |
|  |  |  |  |  |  |  |  |  |

1. **Please provide additional comments or suggestions, if any:**

|  |
| --- |

Based on findings from Delphi Round 1, 6 new conditions are added: (1) skin conditions, (2) chronic pain, (3) allergic rhinitis, (4) gout, (5) other mental health conditions and (6) neurological disorders. Additionally, “physical disability” condition from original list is now split into 2 new conditions: functional limitations and cognitive limitations. The following information is provided for subsequent questions for your reference:

- *Criteria 1: Prevalence of Condition:* ICD-10 codes were mapped to these new conditions based on availability of ICD-10 codes currently used in Polyclinic clusters. We have indicated where an ICD-10 code is not available currently for coding in Polyclinic clusters. Prevalence estimates are calculated based on Polyclinic clusters’ data availability and may not be representative of complete primary care landscape.
- *Criteria 2: Panellists:* Number of panellists in Delphi Round 1 who suggested that the condition in question be included in the expanded list of conditions.
- *Criteria 3: Coverage under CDMP:* Whether or not the condition (any of the included diseases/illnesses) is currently covered under the Chronic Disease Management Program (CDMP).
- *Criteria 4: Relevance in Primary Care Setting:* A condition is considered relevant if it can be appropriately and adequately diagnosed and managed in the primary care setting (based on consensus reached by research team).

**For subsequent questions, please rate each of the chronic conditions in the order of importance (1: limited importance and exclude to 9: critical importance and include) to be included in the final list of chronic conditions to be used for studying multimorbidity in primary care in Singapore.**

1. **Skin conditions with following ICD-10 codes: (1) L70.9 Other acne, (2) L20.8 Other atopic dermatitis, (3) L40.0 Psoriasis vulgaris, (4) L40.8 Other psoriasis**

- *Criteria 1: Prevalence of Condition:* 3%
- *Criteria 2: Panellists:* 6
- *Criteria 3: Coverage under CDMP:* Yes (only Psoriasis)
- *Criteria 4: Relevance in Primary Care Setting:* Relevant

| Limited Importance |  |  |  |  |  |  |  | Critical Importance |
| --- | --- | --- | --- | --- | --- | --- | --- | --- |
| 1 | 2 | 3 | 4 | 5 | 6 | 7 | 8 | 9 |
|  |  |  |  |  |  |  |  |  |
|  |  |  |  |  |  |  |  |  |

1. **Please provide additional comments or suggestions, if any:**

|  |
| --- |

1. **Chronic pain with following ICD-10 codes: (1) Pain*, (2) chronic fatigue*, (3) fibromyalgia*** (*: ICD-10 codes are not used in Polyclinic Coding)

- *Criteria 1: Prevalence of Condition:* *Not available*
- *Criteria 2: Panellists:* 8
- *Criteria 3: Coverage under CDMP:* No
- *Criteria 4: Relevance in Primary Care Setting:* Relevant

| Limited Importance |  |  |  |  |  |  |  | Critical Importance |
| --- | --- | --- | --- | --- | --- | --- | --- | --- |
| 1 | 2 | 3 | 4 | 5 | 6 | 7 | 8 | 9 |
|  |  |  |  |  |  |  |  |  |
|  |  |  |  |  |  |  |  |  |

1. **Please provide additional comments or suggestions, if any:**

|  |
| --- |

1. **Allergic rhinitis with following ICD-10 codes: (1) J30.4 Allergic rhinitis, unspecified**

- *Criteria 1: Prevalence of Condition:* 6%
- *Criteria 2: Panellists:* 1
- *Criteria 3: Coverage under CDMP:* No
- *Criteria 4: Relevance in Primary Care Setting:* Relevant

| Limited Importance |  |  |  |  |  |  |  | Critical Importance |
| --- | --- | --- | --- | --- | --- | --- | --- | --- |
| 1 | 2 | 3 | 4 | 5 | 6 | 7 | 8 | 9 |
|  |  |  |  |  |  |  |  |  |
|  |  |  |  |  |  |  |  |  |

1. **Please provide additional comments or suggestions, if any:**

|  |
| --- |

1. **Gout with following ICD-10 codes: (1) M10.9, Gout, unspecified, (2) M10.99 Gout, unspecified, site unspecified**

- *Criteria 1: Prevalence of Condition:* 3%
- *Criteria 2: Panellists:* 4
- *Criteria 3: Coverage under CDMP:* No
- *Criteria 4: Relevance in Primary Care Setting:* Relevant

| Limited Importance |  |  |  |  |  |  |  | Critical Importance |
| --- | --- | --- | --- | --- | --- | --- | --- | --- |
| 1 | 2 | 3 | 4 | 5 | 6 | 7 | 8 | 9 |
|  |  |  |  |  |  |  |  |  |
|  |  |  |  |  |  |  |  |  |

1. **Please provide additional comments or suggestions, if any:**

|  |
| --- |

1. **Other mental health conditions with following ICD-10 codes: (1) F20.9 Schizophrenia, unspecified, (2) F22.9 Delusional disorder, (3) F29 Unspecified nonorganic psychosis, (4) F31.9 Bipolar affective disorder, unspecified, (5) F48.9 Neurotic disorder, (6) F55.9 Unspecified harmful use of non-dependence producing substance, (7) F99 Mental disorder, not otherwise specified, (8) G47.0 Disorders of initiating and maintaining sleep [insomnias], (9) Z86.5 Personal history of other mental and behavioural disorders, (10) PTSD*, (11) OCD*, (12) Chronic narcotic dependency syndrome/drug abuse*, (13) Personality disorder, (14) Phobia, (15) Somatoform disorders/somatic symptom disorder*, (16) Eating disorders, (17) Alcohol abuse* and (18) Burnout*** (*: ICD-10 codes are not used in Polyclinic Coding)

- *Criteria 1: Prevalence of Condition:* 2%
- *Criteria 2: Panellists:* 9
- *Criteria 3: Coverage under CDMP:* Yes (only Schizophrenia, Bipolar disorder)
- *Criteria 4: Relevance in Primary Care Setting:* Relevant

| Limited Importance |  |  |  |  |  |  |  | Critical Importance |
| --- | --- | --- | --- | --- | --- | --- | --- | --- |
| 1 | 2 | 3 | 4 | 5 | 6 | 7 | 8 | 9 |
|  |  |  |  |  |  |  |  |  |
|  |  |  |  |  |  |  |  |  |

1. **Please provide additional comments or suggestions, if any:**

|  |
| --- |

1. **Neurological disorders with following ICD-10 codes: (1) G40.90 Epilepsy, unspecified, without mention of intractable epilepsy, (2) G20 Parkinson's disease.**

- *Criteria 1: Prevalence of Condition:* 1%
- *Criteria 2: Panellists:* 4
- *Criteria 3: Coverage under CDMP:* Yes
- *Criteria 4: Relevance in Primary Care Setting:* Relevant

| Limited Importance |  |  |  |  |  |  |  | Critical Importance |
| --- | --- | --- | --- | --- | --- | --- | --- | --- |
| 1 | 2 | 3 | 4 | 5 | 6 | 7 | 8 | 9 |
|  |  |  |  |  |  |  |  |  |
|  |  |  |  |  |  |  |  |  |

1. **Please provide additional comments or suggestions, if any:**

|  |
| --- |

1. **Functional Limitation with following ICD-10 codes: (1) G80.9 (Cerebral palsy, unspecified), (2) H91.9 (Hearing loss, unspecified), (3) Q79.9 (Congenital malformation of musculoskeletal system, unspecified), (4) H26.9 (Cataract, unspecified), (5) H54.9 (Unspecified visual impairment), (6) Q89.9 (Congenital malformation, unspecified), (7) Z89.4 (Acquired absence of foot and ankle), (8) Z89.5 (Acquired absence of leg at or below knee), (9) Z89.6 (Acquired absence of leg above knee), (10) M67.99 (Disorder of synovium and tendon, unspecified), (11) M79.89 (Other specified soft tissue disorders, site unspecified), (12) Paraplegia*, (13) Hemiplegia* and (14) Enthesopathy*** (*: ICD-10 codes are not used in Polyclinic Coding)

- *Criteria 1: Prevalence of Condition:* 26%
- *Criteria 2: Panellists:* 8
- *Criteria 3: Coverage under CDMP:* No
- *Criteria 4: Relevance in Primary Care Setting:* Relevant

| Limited Importance |  |  |  |  |  |  |  | Critical Importance |
| --- | --- | --- | --- | --- | --- | --- | --- | --- |
| 1 | 2 | 3 | 4 | 5 | 6 | 7 | 8 | 9 |
|  |  |  |  |  |  |  |  |  |
|  |  |  |  |  |  |  |  |  |

1. **Please provide additional comments or suggestions, if any:**

|  |
| --- |

1. **Cognitive Limitation with following ICD-10 codes: (1) Q90.9 (Down's syndrome, unspecified), (2) F79.9 (Unspecified mental retardation without mention of impairment of behaviour), (3) Autism* and (4) ADHD*** (*: ICD-10 codes are not used in Polyclinic Coding)

- *Criteria 1: Prevalence of Condition:* 0%
- *Criteria 2: Panellists:* 8
- *Criteria 3: Coverage under CDMP:* No
- *Criteria 4: Relevance in Primary Care Setting:* Relevant

| Limited Importance |  |  |  |  |  |  |  | Critical Importance |
| --- | --- | --- | --- | --- | --- | --- | --- | --- |
| 1 | 2 | 3 | 4 | 5 | 6 | 7 | 8 | 9 |
|  |  |  |  |  |  |  |  |  |
|  |  |  |  |  |  |  |  |  |

1. **Please provide additional comments or suggestions, if any:**

|  |
| --- |
