# Supplementary material for: Defining and measuring multimorbidity in primary care in Singapore: Results of an online Delphi study
Source: PLoS One. 2022 Dec 1;17(12):e0278559. doi: 10.1371/journal.pone.0278559 (PMC9714819; doi:10.1371/journal.pone.0278559)
Supplement: S4 Appendix — (DOCX) [file pone.0278559.s004.docx]

**S4 Appendix**. Delphi Round 3 Survey

Thank you for providing your valuable feedback to the questions in Delphi Round 2. Based on the findings of Delphi Round 2 (which we have shared with you in the Round 3 study invite email), we have developed Round 3 survey questions. We would not be asking the questions on which consensus was achieved in Round 2. Therefore, you will be answering fewer questions to mainly deliberate on questions, which did not achieve consensus on the pre-defined criteria (which we have shared with you in the summary findings document). We invite you to answer these questions to facilitate developing a common agreed upon definition of multimorbidity in Singapore. We strongly recommend that you read the summary of findings, your individual response document and multimorbidity (brief information) pdf slides before attempting Round 3 questions.

# Section A (Defining and Operationalizing Multimorbidity)

1. **Please enter your assigned Participant Number here**: ____________

Adopting a comprehensive approach to defining multimorbidity using chronic conditions, based on feedback from Round 1, we shared with you 6 components to define a chronic condition in Round 2. Consensus was achieved for 4 (i.e., duration of condition (71.9% voted yes), impact on patient (81.3% voted yes), management of patient (81.3% voted yes) and recurrent or persistent course of condition (96.9% voted yes)) of these 6 components, and these will be included in the final definition of a chronic condition.

However, consensus was not achieved for the following 2 components: incurable condition and sequelae of condition. With the knowledge of the Round 2 results provided for each of these components, we invite you to vote for these 2 components again in Round 3 so that we can achieve consensus on including/excluding these 2 components from the definition of a chronic condition:

1. [a] Definition of Chronic Condition: **A condition needs to be incurable to be defined as a chronic condition:** *(Note: Incurable is defined as not curable; that cannot be remedied or corrected.)* (65.6% voted ‘Yes’ & 34.5% voted ‘No’ in Round 2)
   1. Yes
   2. No
2. [a] Definition of Chronic Condition: **A condition needs to have a sequela to be defined as a chronic condition:** *(Note: Sequela is defined as any abnormal bodily condition or disease related to or arising from a pre-existing disease; any complication of a disease.)* (62.5% voted ‘Yes’ & 37.5% voted ‘No’ in Round 2).
   1. Yes
   2. No

While panellists agreed that duration of a condition is important in defining a chronic condition, consensus was not achieved on the exact duration to define a condition as chronic. The highest proportion of panellists (50%) voted for the duration of 6 months or more for defining a condition as chronic. Hence, we invite you to deliberate on this question again so that consensus can be achieved.

1. [a] Definition of Chronic Condition: **For a condition to be defined as chronic, the condition should last for:** (6.3% voted 1 month or more, 25% voted 3 months or more, 50% voted 6 months or more and 18.7% voted 12 months or more in Round 2).
   1. 1 month or more -> Go to Question 5.
   2. 3 months or more -> Go to Question 5.
   3. 6 months or more -> Go to Question 6.
   4. 12 months or more -> Go to Question 5.
2. **Please elaborate why you did not choose 6 months or more since majority of the panellists voted for this option previously**: _________________________

[A] Definition of Chronic Condition: Impact on Patient: More than 70% of the panellists voted for impact on the patient to be considered across the following 3 sub-components: limitations in activities of daily living or instrumental activities of daily living or physical disability (96.9%), mortality (75%) and psychological impairment (87.5%). About 43.8% voted for social deprivation to be included, but this item was excluded from the final definition as consensus criteria was not reached. Since the overall consensus criteria for this question of impact on patient was met, the impact on patient would include these 3 components in defining multimorbidity in our context.

[B] Number of conditions in the Multimorbidity List: Based on panellists’ responses in Round 1, the list of conditions was expanded to include 27 conditions. Based on Round 2 voting, majority of the panellists (84.4%) agreed to define multimorbidity based on this proposed list of 27 conditions.

[C] Cut-off for Number of Conditions to Define Multimorbidity: Consensus was achieved with 84.4% of the panellists voting to adopt the cut-off of 3 or more conditions. This cut-off will be incorporated in the final definition of multimorbidity in the local context.

[d] Data Source(s) to Confirm Conditions: Panellists were asked to indicate the most appropriate data source(s) for confirming multimorbidity from patients’, providers’ and health system’s perspective in Round 2. To further move towards a consensus on the primary data source for each of these 3 perspectives, we would like to ask you further questions in Round 3.

1. Majority of the panellists (93.8%) voted for Patient Self-Reported Outcomes as the most appropriate data source for measuring multimorbidity from patients’ perspective (e.g., quality of life, self-rated health, treatment burden etc.). **Do you agree that Patient Self-Reported Outcomes should be the primary recommended data source for measuring multimorbidity from patients’ perspective?**
   1. Yes
   2. No
2. All panellists (100%) voted for Electronic Health Records as the most appropriate data source for measuring multimorbidity from providers’ perspective (e.g., safety, consult time etc.). **Do you agree that Electronic Health Records should be the primary recommended data source for measuring multimorbidity from providers’ perspective?**
   1. Yes
   2. No
3. Majority of the panellists (87.5%) voted for Ministry of Health administrative data as the most appropriate data source for measuring multimorbidity from health system’s perspective. **Do you agree that Ministry of Health administrative data should be the primary recommended data source for measuring multimorbidity from health system’s perspective?** (*Note: Ministry of Health administrative data is defined as a comprehensive database maintained by the Ministry of Health in Singapore, which comprises of the following: health services utilization data, claims data and patient medical records*).
   1. Yes
   2. No
4. [e] Severity of Included Conditions: Severity of chronic conditions is an important factor to consider when defining the *impact* of multimorbidity on outcomes like treatment burden, resource use and associated cost, quality of life etc. However, for estimation of prevalence of multimorbidity at national level (e.g., to track longitudinally), counts of chronic conditions should suffice.

Do you agree with this statement?

Consensus was not achieved with 68.8% agreeing with the above statement.

Some of the panellists suggested that designing or adopting an index to measure multimorbidity burden incorporating severity of included conditions or proxy indicators for severity (e.g., healthcare utilization or polypharmacy etc.) should be included. There was also a suggestion that counts without incorporation of severity may over-estimate the multimorbidity burden. Other suggestions included considerations for longitudinal trajectory of the disease and psychosocial aspects.

Based on the feedback received from the panellists in Round 2, we have revised the approach to describe multimorbidity by adopting a ‘tiered’ strategy, whereby we propose to describe multimorbidity at 2 levels or tiers. Tier 1 will describe multimorbidity using counts of conditions to provide a ‘simple’ description of the prevalence estimate of multimorbidity. Tier 2 will build on Tier 1 and delve deeper into describing multimorbidity by using a locally designed/adapted index to measure multimorbidity incorporating the different parameters highlighted by the panellists, including the severity of conditions. Tier 2 will involve further work and will not be covered in this Delphi study.

With this ‘tiered’ strategy of describing multimorbidity in mind and *focussing on Tier 1 only*, we invite you to answer the following question.

[e] Severity of Included Conditions: Severity of chronic conditions is an important factor to consider when defining the *impact* of multimorbidity on outcomes like treatment burden, resource use and associated cost, quality of life etc. However, for estimation of prevalence of multimorbidity at national level (e.g., to track longitudinally), counts of chronic conditions should suffice.

**Do you agree with this statement?** (68.8% voted ‘Yes’ & 31.2% voted ‘No’ in Round 2)

- 1. Yes
  2. No -> Go to question 10

1. **Please elaborate why you did not vote yes since majority of the panellists voted for this option previously:** _______________________________________

# Section B [Proposed Expanded List of Chronic Conditions]

With a list of chronic conditions for studying multimorbidity in primary care as proposed by international researchers [1], multimorbidity researchers within Singapore developed a context-specific and locally relevant list of chronic conditions for studying multimorbidity in primary care in Singapore, which was shared with you in Delphi Round 2. **Based on Delphi Round 2 findings, 17 conditions (highlighted yellow in the table below) have achieved consensus voting by panellists**, which will be included in the final list of conditions for defining multimorbidity in Singapore. **The remaining 10 conditions will be included in this Round 3 survey** and we invite you to further deliberate and indicate your preference so that consensus can be achieved on these 10 conditions.

(Reference 1: Fortin M, Almirall J, Nicholson K. Development of a research tool to document self-reported chronic conditions in primary care. J Comorb. 2017;7(1):117-123. Published 2017 Nov 9. doi:10.15256/joc.2017.7.122)

Please ***click here*** to access the list of conditions:

| **Expanded List of Conditions [Conditions highlighted yellow have achieved consensus based on voting by Panellists in Round 2]** | | |
| --- | --- | --- |
| **S/N** | **Conditions** | **ICD-10 Codes** |
| 1 | Hyperlipidaemia | E78.5 (Hyperlipidaemia, unspecified) |
| 2 | Hypertension (high blood pressure) | I10 (Essential (primary) hypertension) |
| 3 | Diabetes  (including pre-diabetes) | E09 (Impaired glucose regulation) |
|  |  | E099 (Impaired glucose regulation without complication) |
|  |  | E10.9 (Type 1 diabetes mellitus without complication) |
|  |  | E11.9 (Type 2 diabetes mellitus without complication) |
|  |  | E14.2 (Diabetes mellitus with incipient diabetic nephropathy) |
|  |  | E14.3 (Diabetes mellitus with retinopathy) |
|  |  | E14.31 (Unspecified diabetes mellitus with background retinopathy) |
|  |  | E14.64 (Unspecified diabetes mellitus with hypoglycaemia) |
|  |  | E14.73 (Unspecified diabetes mellitus with foot ulcer due to multiple causes) |
| 4 | Arthritis &/or rheumatoid arthritis | M06.99 (Rheumatoid arthritis, unspecified, site unspecified) |
|  |  | M15.9 (Osteoarthritis (OA) - Generalised) |
|  |  | M19.99 (Arthritis, unspecified, site unspecified) |
| 5 | Obesity | E66.9 (Obesity, unspecified) |
| 6 | Cardiovascular disease (angina, MI, AF, poor circulation of lower limbs) | I25.9 (Chronic ischaemic heart disease, unspecified) |
|  |  | I48 (Atrial fibrillation and flutter) |
|  |  | I70.20 (Atherosclerosis of arteries of extremities, unspecified) |
|  |  | I73.9 (Peripheral vascular disease, unspecified) |
| 7 | Asthma, COPD, or chronic bronchitis | J44.9 (Chronic Obstructive Pulmonary Disease, Unspecified) |
|  |  | J45.9 (Asthma, unspecified) |
| 8 | Chronic hepatitis | K76.9 (Liver disease, unspecified) |
|  |  | Z22.51 (Carrier of viral hepatitis B) |
| 9 | Stomach problem (reflux, heartburn, or gastric ulcer) | K21.9 (Gastro-oesophageal reflux disease without oesophagitis) |
|  |  | K27.9 (Peptic ulcer, unspecified as acute or chronic, without haemorrhage or perforation) |
| 10 | Thyroid disorder | E03.9 (Hypothyroidism, unspecified) |
|  |  | E05.9 (Thyrotoxicosis, unspecified) |
| 11 | Stroke and TIA | G45.9 (Transient cerebral ischaemic attack, unspecified) |
|  |  | I64 (Stroke, not specified as haemorrhage or infarction) |
| 12 | Heart failure (including valve problems or replacement) | I50.0 (Congestive heart failure) |
|  |  | I51.9 (Heart disease, unspecified) |
| 13 | Kidney disease or failure | N03.9 (Unspecified nephritic syndrome, unspecified) |
|  |  | N18.9 (Chronic kidney disease, unspecified) |
| 14 | Depression or anxiety | F32.20 (Severe depressive episode without psychotic symptoms, not specified as arising in the postnatal period) |
|  |  | F32.90 (Depressive episode, unspecified, not specified as arising in the postnatal period) |
|  |  | F41.1 (Anxiety disorder, unspecified) |
| 15 | Chronic urinary problem | N40 (Hyperplasia of prostate), |
|  |  | N39 (Other disorders of urinary system) |
|  |  | N20.9 (Urinary calculus, unspecified) |
|  |  | Incontinence* |
| 16 | Functional Limitation | H91.9 (Hearing loss, unspecified) |
|  |  | Q79.9 (Congenital malformation of musculoskeletal system, unspecified) |
|  |  | G80.9 (Cerebral palsy, unspecified) |
|  |  | H26.9 (Cataract, unspecified) |
|  |  | H54.9 (Unspecified visual impairment) |
|  |  | Q89.9 (Congenital malformation, unspecified) |
|  |  | Z89.4 (Acquired absence of foot and ankle) |
|  |  | Z89.5 (Acquired absence of leg at or below knee) |
|  |  | Z89.6 (Acquired absence of leg above knee) |
|  |  | M67.99 (Disorder of synovium and tendon, unspecified) |
|  |  | M79.89 (Other specified soft tissue disorders, site unspecified) |
|  |  | Paraplegia* |
|  |  | Hemiplegia* |
|  |  | Enthesopathy* |
| 17 | Cognitive Limitation | Q90.9 (Down's syndrome, unspecified) |
|  |  | F79.9 (Unspecified mental retardation without mention of impairment of behaviour) |
|  |  | Autism* |
|  |  | ADHD* |
| 18 | Any cancer in the last 5 years | C80 (Malignant neoplasm without specification of site) |
| 19 | Osteoporosis | M81.99 (Other osteoporosis, site unspecified) |
| 20 | Dementia or Alzheimer's disease | F03 (Unspecified dementia) |
| 21 | Colon problem (irritable bowel) | K58.9 (Irritable bowel syndrome without diarrhoea) |
| 22 | Skin Conditions | L70.9 (Other Acne) |
|  |  | L20.8 (Other Atopic Dermatitis) |
|  |  | L40.0 (Psoriasis Vulgaris) |
|  |  | L40.8 (Other Psoriasis) |
| 23 | Chronic Pain | Pain* |
|  |  | Chronic Fatigue* |
|  |  | Fibromyalgia* |
| 24 | Allergic rhinitis | J30.4 (Allergic rhinitis, unspecified) |
| 25 | Gout | M10.9 (Gout, unspecified) |
|  |  | M10.99 (Gout, unspecified, site unspecified) |
| 26 | Other Mental Health Conditions | F20.9 (Schizophrenia, unspecified) |
|  |  | F22.9 (Delusional disorder) |
|  |  | F29 (Unspecified nonorganic psychosis) |
|  |  | F31.9 (Bipolar affective disorder, unspecified) |
|  |  | F48.9 (Neurotic disorder) |
|  |  | F55.9 (Unspecified harmful use of non-dependence producing substance) |
|  |  | F99 (Mental disorder, not otherwise specified) |
|  |  | G47.0 (Disorders of initiating and maintaining sleep [insomnias]) |
|  |  | Z86.5 (Personal history of other mental and behavioural disorders) |
|  |  | PTSD* |
|  |  | OCD* |
|  |  | Chronic narcotic dependency syndrome/drug abuse* |
|  |  | Personality disorder* |
|  |  | Phobia* |
|  |  | Somatoform disorders/somatic symptom disorder* |
|  |  | Eating disorders* |
|  |  | Alcohol abuse* |
|  |  | Burnout* |
| 27 | Neurological Disorders | G40.90 Epilepsy, unspecified, without mention of intractable epilepsy |
|  |  | G20 Parkinson's disease |
|  | *: ICD-10 codes are not used in Polyclinic Coding | |

Though majority of the panellists (84.4%) agreed to include the above 27 conditions in the list for defining multimorbidity (Section A, Point B), for 10 of these 27 conditions, many panellists have voted these conditions as important but not critical (rating between 4 to 6) in Round 2. While it is established from Round 2 that panellists consider these conditions as important, moving forward, we would like to seek your inputs on which of these 10 conditions should be included in the final list of conditions for defining multimorbidity. Based on your vote in Round 3, the **final** list of conditions for defining multimorbidity can be as low as 17 or as high as 27.

**We invite you to indicate your preference (as yes/no) for including each of these 10 conditions in the final list of conditions for defining multimorbidity.**

1. **Should obesity with following ICD codes: (1) E66.9 (Obesity, unspecified) be included in the final list of conditions for defining multimorbidity?** (67.7% voted critical, 29.0% voted important not critical, 3.2% voted limited importance)
   1. Yes -> Go to Question 2.
   2. No -> Go to Question 3.
2. **Please elaborate why you chose to vote yes for including obesity in the final list of conditions for defining multimorbidity**: _________________
3. **Please elaborate why you chose to vote no for including obesity in the final list of conditions for defining multimorbidity**: __________________
4. **Should chronic hepatitis with following ICD-10 codes: (1) K76.9 (Liver disease, unspecified), (2) Z22.51 (Carrier of viral hepatitis B) be included in the final list of conditions for defining multimorbidity?** (48.4% voted critical, 45.2% voted important not critical, 6.5% voted limited importance)
   1. Yes -> Go to Question 5.
   2. No -> Go to Question 6.
5. **Please elaborate why you chose to vote yes for including chronic hepatitis in the final list of conditions for defining multimorbidity:** _________________
6. **Please elaborate why you chose to vote no for including chronic hepatitis in the final list of conditions for defining multimorbidity:** __________________
7. **Should stomach problem (reflux, heartburn, or gastric ulcer) with following ICD codes: (1) K21.9 (Gastro-oesophageal reflux disease without oesophagitis), (2) K27.9 (Peptic ulcer, unspecified as acute or chronic, without haemorrhage or perforation) be included in the final list of conditions for defining multimorbidity?** (22.6% voted critical, 54.8% voted important not critical, 22.6% voted limited importance)
   1. Yes -> Go to Question 8.
   2. No -> Go to Question 9.
8. **Please elaborate why you chose to vote yes for including stomach problem (reflux, heartburn, or gastric ulcer) in the final list of conditions for defining multimorbidity**: _________________
9. **Please elaborate why you chose to vote no for including stomach problem (reflux, heartburn, or gastric ulcer) in the final list of conditions for defining multimorbidity**: __________________
10. **Should thyroid disorder with following ICD-10 codes: (1) E03.9 (Hypothyroidism, unspecified), (2) E05.9 (Thyrotoxicosis, unspecified) be included in the final list of conditions for defining multimorbidity?** (48.4% voted critical, 51.6% voted important not critical, 0.0% voted limited importance)
    1. Yes -> Go to Question 11.
    2. No -> Go to Question 12.
11. **Please elaborate why you chose to vote yes for including thyroid disorder in the final list of conditions for defining multimorbidity**: _________________
12. **Please elaborate why you chose to vote no for including thyroid disorder in the final list of conditions for defining multimorbidity:** __________________
13. **Should chronic urinary problem with following ICD-10 codes: (1) N40 (Hyperplasia of prostate), N39 (Other disorders of urinary system), N20.9 (Urinary calculus, unspecified) and Incontinence* be included in the final list of conditions for defining multimorbidity?** (*: ICD-10 codes are not used in Polyclinic Coding) (38.7% voted critical, 61.3% voted important not critical, 0.0% voted limited importance)
    1. Yes -> Go to Question 14.
    2. No -> Go to Question 15.
14. **Please elaborate why you chose to vote yes for including chronic urinary problem in the final list of conditions for defining multimorbidity**: _________________
15. **Please elaborate why you chose to vote no for including chronic urinary problem in the final list of conditions for defining multimorbidity:** __________________
16. **Should colon problem (irritable bowel) with following ICD-10 codes: (1) K58.9 (Irritable bowel syndrome without diarrhoea) be included in the final list of conditions for defining multimorbidity?** (38.7% voted critical, 58.1% voted important not critical, 3.2% voted limited importance)
    1. Yes -> Go to Question 17.
    2. No -> Go to Question 18.
17. **Please elaborate why you chose to vote yes for including colon problem (irritable bowel) in the final list of conditions for defining multimorbidity**: _________________
18. **Please elaborate why you chose to vote no for including colon problem (irritable bowel) in the final list of conditions for defining multimorbidity**: __________________
19. **Should skin conditions with following ICD-10 codes: (1) L70.9 Other acne, (2) L20.8 Other atopic dermatitis, (3) L40.0 Psoriasis vulgaris, (4) L40.8 Other psoriasis be included in the final list of conditions for defining multimorbidity?** (45.2% voted critical, 45.2% voted important not critical, 9.7% voted limited importance)
    1. Yes -> Go to Question 20.
    2. No -> Go to Question 21.
20. **Please elaborate why you chose to vote yes for including skin conditions in the final list of conditions for defining multimorbidity**: _________________
21. **Please elaborate why you chose to vote no for including skin conditions in the final list of conditions for defining multimorbidity:** __________________
22. **Should chronic pain with following ICD-10 codes: (1) Pain*, (2) chronic fatigue*, (3) fibromyalgia*be included in the final list of conditions for defining multimorbidity?** (*: ICD-10 codes are not used in Polyclinic Coding) (61.3% voted critical, 35.5% voted important not critical, 3.2% voted limited importance)
    1. Yes -> Go to Question 23.
    2. No -> Go to Question 24.
23. **Please elaborate why you chose to vote yes for including chronic pain in the final list of conditions for defining multimorbidity**: _________________
24. **Please elaborate why you chose to vote no for including chronic pain in the final list of conditions for defining multimorbidity**: __________________
25. **Should allergic rhinitis with following ICD-10 codes: (1) J30.4 Allergic rhinitis, unspecified be included in the final list of conditions for defining multimorbidity**? (16.1% voted critical, 38.7% voted important not critical, 45.2% voted limited importance)
    1. Yes -> Go to Question 26.
    2. No -> Go to Question 27.
26. **Please elaborate why you chose to vote yes for including allergic rhinitis in the final list of conditions for defining multimorbidity**: _________________
27. **Please elaborate why you chose to vote no for including allergic rhinitis in the final list of conditions for defining multimorbidity**: __________________
28. **Should gout with following ICD-10 codes: (1) M10.9, Gout, unspecified, (2) M10.99 Gout, unspecified, site unspecified be included in the final list of conditions for defining multimorbidity**? (51.6% voted critical, 38.7% voted important not critical, 9.7% voted limited importance)
    1. Yes -> Go to Question 29.
    2. No -> Go to Question 30.
29. **Please elaborate why you chose to vote yes for including gout in the final list of conditions for defining multimorbidity**: _________________
30. **Please elaborate why you chose to vote no for including gout in the final list of conditions for defining multimorbidity:** __________________

Thank you for completing the survey. We are very grateful for your feedback and look forward to your continuous participation.
