# Supplementary material for: Defining and measuring multimorbidity in primary care in Singapore: Results of an online Delphi study
Source: PLoS One. 2022 Dec 1;17(12):e0278559. doi: 10.1371/journal.pone.0278559 (PMC9714819; doi:10.1371/journal.pone.0278559)
Supplement: S5 Appendix — (DOCX) [file pone.0278559.s005.docx]

**S5. Appendix**. Finalised list of chronic conditions for defining multimorbidity

| **S/N** | **Conditions^1^** | **ICD-10 Codes** |
| --- | --- | --- |
| 1 | Any cancer in the last 5 years | C80 (Malignant neoplasm without specification of site) |
| 2 | Arthritis &/or rheumatoid arthritis | M06.99 (Rheumatoid arthritis, unspecified, site unspecified) |
|  |  | M15.9 (Osteoarthritis (OA) - Generalised) |
|  |  | M19.99 (Arthritis, unspecified, site unspecified) |
| 3 | Asthma, COPD, or chronic bronchitis | J44.9 (Chronic Obstructive Pulmonary Disease, Unspecified) |
|  |  | J45.9 (Asthma, unspecified) |
| 4 | Cardiovascular disease (angina, MI, AF, poor circulation of lower limbs) | I25.9 (Chronic ischaemic heart disease, unspecified) |
|  |  | I48 (Atrial fibrillation and flutter) |
|  |  | I70.20 (Atherosclerosis of arteries of extremities, unspecified) |
|  |  | I73.9 (Peripheral vascular disease, unspecified) |
| 5 | Chronic hepatitis | K76.9 (Liver disease, unspecified) |
|  |  | Z22.51 (Carrier of viral hepatitis B) |
| 6 | Chronic Pain | Pain* |
|  |  | Chronic Fatigue* |
|  |  | Fibromyalgia* |
| 7 | Chronic urinary problem | N40 (Hyperplasia of prostate), |
|  |  | N39 (Other disorders of urinary system) |
|  |  | N20.9 (Urinary calculus, unspecified) |
|  |  | Incontinence* |
| 8 | Cognitive Limitation | Q90.9 (Down's syndrome, unspecified) |
|  |  | F79.9 (Unspecified mental retardation without mention of impairment of behaviour) |
|  |  | Autism* |
|  |  | ADHD* |
| 9 | Depression or anxiety | F32.20 (Severe depressive episode without psychotic symptoms, not specified as arising in the postnatal period) |
|  |  | F32.90 (Depressive episode, unspecified, not specified as arising in the postnatal period) |
|  |  | F41.1 (Anxiety disorder, unspecified) |
| 10 | Dementia or Alzheimer's disease | F03 (Unspecified dementia) |
| 11 | Diabetes  (including pre-diabetes) | E09 (Impaired glucose regulation) |
|  |  | E099 (Impaired glucose regulation without complication) |
|  |  | E10.9 (Type 1 diabetes mellitus without complication) |
|  |  | E11.9 (Type 2 diabetes mellitus without complication) |
|  |  | E14.2 (Diabetes mellitus with incipient diabetic nephropathy) |
|  |  | E14.3 (Diabetes mellitus with retinopathy) |
|  |  | E14.31 (Unspecified diabetes mellitus with background retinopathy) |
|  |  | E14.64 (Unspecified diabetes mellitus with hypoglycaemia) |
|  |  | E14.73 (Unspecified diabetes mellitus with foot ulcer due to multiple causes) |
| 12 | Functional Limitation | H91.9 (Hearing loss, unspecified) |
|  |  | Q79.9 (Congenital malformation of musculoskeletal system, unspecified) |
|  |  | G80.9 (Cerebral palsy, unspecified) |
|  |  | H26.9 (Cataract, unspecified) |
|  |  | H54.9 (Unspecified visual impairment) |
|  |  | Q89.9 (Congenital malformation, unspecified) |
|  |  | Z89.4 (Acquired absence of foot and ankle) |
|  |  | Z89.5 (Acquired absence of leg at or below knee) |
|  |  | Z89.6 (Acquired absence of leg above knee) |
|  |  | M67.99 (Disorder of synovium and tendon, unspecified) |
|  |  | M79.89 (Other specified soft tissue disorders, site unspecified) |
|  |  | Paraplegia* |
|  |  | Hemiplegia* |
|  |  | Enthesopathy* |
| 13 | Gout | M10.9 (Gout, unspecified) |
|  |  | M10.99 (Gout, unspecified, site unspecified) |
| 14 | Heart failure (including valve problems or replacement) | I50.0 (Congestive heart failure) |
|  |  | I51.9 (Heart disease, unspecified) |
| 15 | Hyperlipidaemia | E78.5 (Hyperlipidaemia, unspecified) |
| 16 | Hypertension (high blood pressure) | I10 (Essential (primary) hypertension) |
| 17 | Kidney disease or failure | N03.9 (Unspecified nephritic syndrome, unspecified) |
|  |  | N18.9 (Chronic kidney disease, unspecified) |
| 18 | Neurological Disorders | G40.90 Epilepsy, unspecified, without mention of intractable epilepsy |
|  |  | G20 Parkinson's disease |
| 19 | Obesity | E66.9 (Obesity, unspecified) |
| 20 | Osteoporosis | M81.99 (Other osteoporosis, site unspecified) |
| 21 | Other Mental Health Conditions | F20.9 (Schizophrenia, unspecified) |
|  |  | F22.9 (Delusional disorder) |
|  |  | F29 (Unspecified nonorganic psychosis) |
|  |  | F31.9 (Bipolar affective disorder, unspecified) |
|  |  | F48.9 (Neurotic disorder) |
|  |  | F55.9 (Unspecified harmful use of non-dependence producing substance) |
|  |  | F99 (Mental disorder, not otherwise specified) |
|  |  | G47.0 (Disorders of initiating and maintaining sleep [insomnias]) |
|  |  | Z86.5 (Personal history of other mental and behavioural disorders) |
|  |  | PTSD* |
|  |  | OCD* |
|  |  | Chronic narcotic dependency syndrome/drug abuse* |
|  |  | Personality disorder* |
|  |  | Phobia* |
|  |  | Somatoform disorders/somatic symptom disorder* |
|  |  | Eating disorders* |
|  |  | Alcohol abuse* |
|  |  | Burnout* |
| 22 | Stroke and TIA | G45.9 (Transient cerebral ischaemic attack, unspecified) |
|  |  | I64 (Stroke, not specified as haemorrhage or infarction) |
| 23 | Thyroid disorder | E03.9 (Hypothyroidism, unspecified) |
|  |  | E05.9 (Thyrotoxicosis, unspecified) |
|  | 1: Consensus was not achieved for the following 3 conditions which are not included under this recommended finalized list of 23 conditions for defining multimorbidity: 1) Stomach problem (reflux, heartburn or gastric ulcer), 2) Colon problem (irritable bowel) and 3) Skin conditions.  *: ICD-10 codes are not used in Polyclinic Coding | |
