# Supplementary material for: Defining and measuring multimorbidity in primary care in Singapore: Results of an online Delphi study
Source: PLoS One. 2022 Dec 1;17(12):e0278559. doi: 10.1371/journal.pone.0278559 (PMC9714819; doi:10.1371/journal.pone.0278559)
Supplement: Attachment — Submitted filename: Response to Reviewers.docx [file pone.0278559.s006.docx]

Prof (Dr) Gerald Koh, MD, PhD

Clinical Director and Head, Future Primary Care (FPC)

MOH Office for Healthcare Transformation

1 Maritime Square, #12-10, Harbourfront Centre

Singapore 099253

[Gerald_koh@moht.com.sg](mailto:Gerald_koh@moht.com.sg)

Dec 14, 2021

Re: Resubmission of manuscript *Defining and measuring multimorbidity in primary care in Singapore: results of an online Delphi study,* Manuscript ID PONE-D-21-28758

Dear Editor,

Greetings.

Thank you so much for seeing merit in our manuscript, entitled “Defining and measuring multimorbidity in primary care in Singapore: results of an online Delphi study,” and giving us the opportunity to revise it further. We really appreciate the feedback we have received.

We once again want to thank you and the reviewers for taking the time and effort to carefully read our work and provide us encouraging feedback, with constructive suggestions to work on. We believe that the manuscript is significantly improved after making the suggested amendments and we hope that it meets your expectations as well.

The revision has been developed in consultation with all co-authors, and each author has given approval to the final form of this revision. Changes made in the manuscript are marked using track changes in the “Revised Manuscript with Track Changes”. We have uploaded this track-changed copy along with a clean copy designated as “Manuscript”, revised figure 1 (study flowchart), and S1 Appendix. CREDES checklist (updated page numbers) for your kind perusal. We have no conflicts of interest to declare.

The datasets used and analysed during the current study are available from the corresponding author on reasonable request. The datasets generated and/or analysed during the current study are not publicly available because they may contain potentially sensitive information related to the views shared by the Delphi panellists under anonymity. Moreover, prior consent was not sought from Delphi panellists to share the data containing their responses outside of the study team.

**Please find below the point-by-point response to all the comments by the Reviewers as required**.

Thank you for your kind consideration.

Sincerely,

Prof (Dr) Gerald Koh, MD, PhD

Clinical Director and Head, Future Primary Care (FPC), MOH Office for Healthcare Transformation

Professor, Saw Swee Hock School of Public Health, National University of Singapore

**POINT-BY-POINT RESPONSE TO COMMENTS BY REVIEWERS.**

Please take note that the page and line numbers mentioned in the responses below are referring to the revised version of the manuscript (marked copy with track-changes).

**Editor Comments.**

Comment: Thank you for submitting your manuscript to PLOS ONE. After careful consideration, we feel that it has merit but does not fully meet PLOS ONE’s publication criteria as it currently stands. Therefore, we invite you to submit a revised version of the manuscript that addresses the points raised during the review process.

Response: Thank you so much for seeing merit in our manuscript and giving us the opportunity to revise it. We have tried to address the reviewers’ comments and made amendments as required. Hopefully, the revised manuscript will meet your standards.

**Reviewer 1:**

Comment 1: This manuscript covers a current and valuable topic in chronic condition care: what is meant by multimorbidity? The key reasons for defining multimorbidity is to facilitate understanding the prevalence of multimorbidity, the health impact and provide targets for clinical improvement and research studies. This study aims to define multimorbidity for a primary care audience in Singapore.

Response: Thank you so much for your positive and encouraging feedback on our manuscript.

Comment 2: The authors’ rationale for the narrow focus of their multimorbidity definition is not well-explained in the manuscript nor why the authors think a new definition from primary care in Singapore is needed compared to adapting a current definition. The manuscript should explain why the authors think their definition is needed and what the implications for its use are. This should be better articulated in the intro and discussion. The intro explains why a universal definition is needed, but the paper doesn’t offer that or is this a first step towards that goal?

Response: Thank you for highlighting this, to address this comment, we have included a description in the revised manuscript detailing our rationale and justification for focussing on the primary care setting.

Please find copied below the added text for reviewer’s kind reference:

“*It is important to describe multimorbidity within a specified setting as the prevalence of multimorbidity has been reported to vary across different settings. [10] Moreover, previous studies exploring multimorbidity have also adopted such a setting-specific approach. [18, 19] With this setting-specific approach, primary care is aptly suited to provide patient-centred holistic care for patients with multiple chronic conditions based on the tenets of continuity, coordination and comprehensiveness. [20] Additionally, it is established that management of multiple chronic diseases by interventions within primary care setting are more cost-effective as compared to other care settings. [21] Hence, for the current study, we focussed on developing a consensus-derived definition of multimorbidity within the primary care setting*.”

We articulated our rationale and justification for focussing on the primary care setting within Singapore to develop a consensus-derived definition of multimorbidity and the potentiation implications of this. To address the reviewer’s comment, we have revised the introduction section to further clarify the rationale and justification for focussing on the primary care setting within Singapore and also the potential implications.

-	Page – 4, line numbers 73 to 81, 82 to 97.

Comment 3: The paper would be stronger if it more clearly compared their new definition against existing definitions (3 vs 2 conditions, out of a list of 27, and which conditions included) and what these means clinically. Would some patients have multimorbidity form the new definition vs not another, and does this matter? Consider adding more definitions – US AHRQ definition is missing. I saw WHO, European and Australia mentioned but the comparisons were limited. How might it change clinical care to have a 3 vs 2 cut off, to have a different list of conditions or different subgroupings of conditions?

Response: Thank you so much for this useful suggestion. To address this, we have explicitly compared our new recommended definition against existing definitions in the revised manuscript, compared and provided implications of recommending a threshold of 3 or more as compared to 2 or more in existing literature, added a comparative description of our finalised list of 23 conditions & the type of conditions included with the existing literature. Additionally, we have also provided implications arising from these comparisons as well in the revised manuscript. We have also added the US AHRQ definition of multiple chronic conditions in the revised manuscript. Lastly, we have provided the change in clinical care needed while using a cut-off of 3 or more as compared to 2 or more in the revised manuscript.

The change is reflected in the following sections of the revised manuscript:

-	Under introduction section, page – 3, lines 56 to 58

-	Under discussion section, pages – 27 to 29, line numbers – 406 to 4321, 444 to 472.

Comment 4: The paper would also be stronger if the authors explained why specific conditions were included and why they were grouped in the first round. Why were depression and anxiety grouped together but bipolar was not included with them and rather with “other”? Why was asthma with COPD and not separate? Some of the groupings are for very similar conditions but others could be argued as separate chronic conditions – the rationale for these groupings pre-Delphi needs to be made clear. Also, were inflammatory bowel diseases included? I only saw IBS. What was the rationale for the limited conditions included at the beginning? Please explain how these were chosen. Also, address more explicitly “risk factors” vs conditions (how were obesity, hyperlipidemia, etc handled)

Response: Thank you so much for this comment, to address this we have provided a detailed description of the pre-Delphi list of conditions. The preliminary list of conditions used as a starting point for our Delphi study was based on the proposed list of 20 conditions provided by Fortin and colleagues (2017). Along with providing a reference for the detailed methodology of development of this proposed list of conditions in the revised manuscript, we have also provided a short summary of the development process within the revised manuscript. Subsequently, this proposed list of chronic conditions was modified by local researchers from Singapore to increase local primary care relevance. This included adding of pre-diabetes and functional disability. We used this modified version of the proposed list by Fortin and colleagues in our Delphi study.

We have copied the added description here for reviewer’s kind reference:

“*This preliminary list of chronic conditions used as a starting point in the current study was conceptualised based on the originally proposed list of 20 chronic conditions by Fortin and colleagues [37] This list of 20 conditions was derived from a scoping review of 44 publications with a total of 131 short-listed conditions. The short-listed conditions comprised of a diverse combination of diseases (e.g., myocardial infarction), risk factors (e.g., hypertension), symptoms (e.g., faints) and categories of conditions (e.g., respiratory problems). The proposed list of 20 conditions was derived from the 131 short-listed conditions based on the following selection criteria: relevance in primary care setting, impact on the patient, frequency of reporting of conditions in existing studies and prevalence in primary care patients. The grouping together of certain conditions like angina, myocardial infarction, atrial fibrillation etc. under the category of ‘cardiovascular disease’ was based on such conditions affecting the same body system. The detailed methodology is described elsewhere. [37] The list proposed by Fortin et al. [37] was modified to make it suitable for use in primary care setting in Singapore with inclusion of pre-diabetes under the chronic condition of ‘diabetes’ and also inclusion of ‘physical disability’ in the modified list. [16] Throughout the iterative process of deriving the finalised list of conditions for defining multimorbidity, the total conditions included in the list at all times was more than 12, which is the recommended value for preventing underestimation of multimorbidity prevalence. [10]*”

To address the point on providing more information on distinction of “risk factors” vs “conditions” and how they were handled, we have added new text in the discussion section. In summary, the preliminary list of conditions used as a starting point comprised of diseases, risk factors and symptoms. Additionally, the finalised list of conditions based on our Delphi study also comprised of all 3 categories of diseases, risk factors and symptoms. Please refer below for the added text in the revised discussion section:

“*Willadsen et al [17] summarized the evidence on multimorbidity frameworks by categorizing the included conditions into three categories of diseases, risk factors and symptoms. They concluded that while it is important to include risk factors and symptoms along with diseases within the list of conditions for describing multimorbidity, existing literature mainly included diseases in such lists. Risk factors were included by few studies and symptoms were included by even fewer. Out of the most frequently reported diseases, risk factors and symptoms included in such lists, our finalised list of conditions is inclusive of all the diseases and most of the risk factors and symptoms. Our finalized list comprises diseases like diabetes, cardiovascular diseases, stroke etc., risk factors like hypertension, hyperlipidaemia, obesity etc., and symptoms like chronic pain, hearing loss, incontinence etc. The potential implication of inclsuding risk factors and symptoms in our finalised list is that the multimorbidity prevalence estimated would be higher as compared to including diseases only. [51]*”

The change is reflected in the following:

-	Under methods section, pages -8-9 , line numbers- 179-196.

-	Under discussion section, pages – 29-30, line numbers -473-485.

Comment 5: Also, if they explained why specific conditions are needed for a definition of multimorbidity when they also give a cut off for overall number of conditions and a definition of chronic condition.

Response: Thank you for this query. We followed the established recommendations for developing an operational definition of multimorbidity, whereby it is recommended that these 2 major components (i.e., a threshold/cut-off and list of conditions) be specified. (1) This is aligned with existing systematic reviews on multimorbidity in which the most common sources of variation are number of conditions included in the list of conditions and the chosen threshold. (2-4) Hopefully, this clarified the rationale for including both threshold and list of conditions as constituting components of our consensus-derived definition of multimorbidity.

References:

1.	Fortin, Martin, José Almirall, and Kathryn Nicholson. "Development of a research tool to document self-reported chronic conditions in primary care." (2017): 117-123.

2.	Fortin M, Stewart M, Poitras ME, Almirall J, Maddocks H. A systematic review of prevalence studies on multimorbidity: toward a more uniform methodology. Ann Fam Med. 2012 Mar-Apr;10(2):142-51. doi: 10.1370/afm.1337. PMID: 22412006; PMCID: PMC3315131.

3.	Violan C, Foguet-Boreu Q, Flores-Mateo G, Salisbury C, Blom J, Freitag M, Glynn L, Muth C, Valderas JM. Prevalence, determinants and patterns of multimorbidity in primary care: a systematic review of observational studies. PloS one. 2014 Jul 21;9(7):e102149.

4.	Pati S, Swain S, Hussain MA, van den Akker M, Metsemakers J, Knottnerus JA, Salisbury C. Prevalence and outcomes of multimorbidity in South Asia: a systematic review. BMJ Open. 2015 Oct 7;5(10):e007235. doi: 10.1136/bmjopen-2014-007235. PMID: 26446164; PMCID: PMC4606435.

Comment 6: The paper is lacking a limitations section. Only one limitation is mentioned within a strengths section. One major limitation is that the conditions are those considered important for a Singaporean population. I think the answers on which conditions matter for multimorbidity would be different in a different population (IBS and GERD might be important in the US, for instance) and that needs to be stated. Also, are there any limitations to the sample? The sample seemed young and mostly male.

Response: Thank you so much for this comment. To address this, we have expanded the limitations section in the revised manuscript, included it as a separate para from the strengths section and incorporated the generalisability considerations kindly highlighted by the reviewer.

We have copied the added text here for reviewer’s kind reference:

“*Our study has several limitations. Certain components and chronic conditions did not reach a consensus rating at the end of Delphi round 3. As mentioned above, our finalized list did not include the following three conditions due to lack of consensus: stomach problems, colon problems and skin conditions. Comparing our findings with existing literature, similar exclusions are observed across multiple studies. [5, 8, 25-27, 50] Moreover, locally reported standardised prevalence estimates in primary care setting for these conditions are relatively lower as compared to other conditions within the finalised list. [16] Therefore, the impact of not including these three conditions on prevalence estimation using our consensus-derived definition of multimorbidity would likely be minimal. Our approach of conducting three Delphi rounds is in accordance with the Delphi methodology recommendations, taking into consideration principle of diminishing returns, potential participant fatigue and steep drop-out rates with higher number of rounds. [59, 60] Since our study primarily aimed to gain consensus on defining multimorbidity within Singapore’s primary care setting, the generalisability of our findings would be limited to similar settings as Singapore. Within Singapore, the generalisability of our findings may be potentially affected by the representativeness of our sample with majority of the panellists being male and relatively younger. Another limitation inherent in the Delphi survey technique is that panellists do not get the opportunity for elaborating their shared views. However, this should not majorly impact our findings considering the focussed and clearly defined scope of work on which consensus was sought*.”

The change is reflected under the discussion section, page 33, lines – 555 to 574.

Comment 7: For Table 5, I recommend putting a column with a check to mark that the condition was included in the final list of conditions. This would improve reading clarity and this important result should be in a main table,. Not just the Appendix.

Response: Thank you so much for this useful suggestion. To incorporate this, we have included an additional column with a check to mark the conditions included in the final list of conditions in Table 5 in the revised manuscript.

The change is reflected in revised Table 5 on pages 23-25.

Comment 8: This is a valuable topic and the paper would add more value to the literature with more clearly stated rationale for needing a Singaporean primary care-based definition (implications for use) and comparisons to current definitions including differences in clinical implications.

Response: Thank you so much for appreciating our work and providing us with constructive feedback to further improve our manuscript. We have addressed both these points raised, i.e., providing rationale for needing a Singaporean primary care based definition (implications for use) and comparisons to current definitions including differences in clinical implications. Please refer to your responses to comments 2 and 3 above, respectively.

**Reviewer 2:**

Comment 1: Thank you for this interesting article. Multimorbidity is an increasingly important topic. In this respect, this work represents a valuable contribution in this field. The article is very well structured, the goal is clearly described and the procedure is presented transparently. On the positive side, you used the CREDES checklist to report the results.

Response: Thank you so much for your positive and encouraging feedback on our manuscript.

Comment 2: Abstract: p. 2 (row 31-32) The increasing response rate is irritating. Even if it is explained later from the flowchart. Maybe the specification like this: 61.7% (37/40), 86.5% (32/37) and 93.8% (30/32)?

Response: Thank you so much for this comment. To address this, we have included the following numbers in the abstract as kindly suggested by the reviewer: 61.7% (37/60), 86.5% (32/37) and 93.8% (30/32).

The change is reflected in the following section of the revised manuscript:

-	Page-2, line number 31.

Comment 3: p.4 (row 71-72): What exactly do you mean by "to improve outcomes of patients with multimorbidity?" Please explain in more detail.

Response: Thank you for this query. To elaborate, this text in the introduction section was highlighting the practical implications of developing a consensus-derived definition of multimorbidity. This standardised approach of defining multimorbidity is a pre-requisite or starting point to further advance our efforts to study different aspects of multimorbidity like its impact on patients’ healthcare utilisation and associated costs, explore commonly co-occurring conditions in patients with multimorbidity and so forth. This in turn would guide/support the development of locally relevant clinical practice recommendations to help clinicians better manage patients with multimorbidity, ensure continuity of care and improve patient outcomes like quality of life and prevent adverse outcomes related to polypharmacy. We hope this clarifies our intention of using “to improve outcomes of patients with multimorbidity”. To improve the clarity as suggested, we have incorporated this detailed explanation in the revised manuscript with the relevant references.

The change is reflected in the following section of the revised manuscript:

-	Pages-4-5, line numbers 87 to 93.

Comment 4: p.3 (row 47): In my opinion, source 4 does not show the named spread (“it ist common….”) of multimorbidity.

Response: Thank you for this comment, as kindly suggested by the reviewer, we have removed reference 4 from this section of the revised manuscript.

The change is reflected on page – 3, line – 47 of the revised manuscript.

Comment 5: p.7 (row159-160): You gave the participants literature so that they would receive unbiased and new ideas. But aren't the ideas then influenced by the literature? Or have I misunderstood that?

Response: Thank you for this query. To further clarify our intention of presenting background information before round 2 and not before round 1, we have provided our explanation below for reviewer’s kind reference:

Round 1 was qualitative with open-ended questions for new idea generation from the panellists (which we may have not incorporated in our preliminary questions/documents), hence we did not share any material/information to ensure panellists share their unbiased ideas. By unbiased ideas, we meant our sharing should not limit the panellists from sharing new/alternative views. The main goal/outcome from round 1 was for us to refine/amend our closed-ended survey questions for round 2 & 3, which were quantitative. For round 2, since the intention was to gain consensus on defined closed-ended questions by panellist voting, we gave basic background information on multimorbidity to enable panellists to vote in case they may not be familiar with some concepts and related heterogeneity in existing literature. To further add, the background information slide deck provided basic information on such concepts and related heterogeneity in existing literature in a neutral manner, which would not have biased their views in any manner. Therefore, while it was prudent to not share background information before round 1, it was relevant to share background information before round 2. Hope this clarifies our chosen approach to sharing background information.

Comment 6: p.9 (row179): “the most common elements” is somewhat unspecific. How many times was it mentioned?

Response: Thank you so much for this query. To address this, we have added the number of respondents who responded with different identified elements under definition of chronic condition in the revised manuscript to explicitly report the frequency of reporting.

The change is reflected on page – 11, lines 222 to 225 of the revised manuscript.

Comment 7: p.9 (row 185): “Most panallists” is also unspecific. How many?

Response: Thank you so much for this query. To address this, we have added the number of respondents who responded with different identified elements under definition of multimorbidity in the revised manuscript to explicitly report the frequency of reporting.

The change is reflected on pages – 11, lines 227 to 234 of the revised manuscript.

Comment 8: p. 16: Please adjust the size of table 4 to all other tables.

Response: Noted, we have adjusted the size of Table 4 to align with all other tables in the revised manuscript.

The change is reflected on page – 19.

Comment 9: p.26: (row429-430): “enabled participants not to be aware of each other’s responses,” But the results of the previous rounds were reflected in them, right?

Response: Thank you for this query. To elaborate, by maintaining pseudo-anonymity, participants were not aware of each other’s individual responses. In other words, they would not be able to attribute one particular response to one particular Delphi panellist. Aligned with the methodology of Delphi study, we did share the results from previous rounds, but as mentioned in the original manuscript, “a summary of findings of that round” was shared with the Delphi panellists. With aggregate or summary estimates, it is impossible to ascertain which panellist voted what. Additionally, only one’s own individual response was shared with each Delphi panellist (not other Delphi panellists’ individual responses). Hope this clarifies the raised point.

Comment 10: Discussion: Please detail to the point "Measuring the burden of multimorbidity". This was raised in Delphi and shown for all rounds. However, it does not appear in the discussion or in the conclusion.

Response: Thank you so much for this comment. To address this comment, we have added the discussion on the point of “Measuring the burden of multimorbidity” in the discussion section of the revised manuscript.

We have copied the added text in the discussion section here for reviewer’s kind reference:

“*The Delphi panellists recommended that burden of multimorbidity be measured from multiple perspectives (e.g., patient, provider, health system or a combination of these). Based on consensus voting, the most appropriate data source for measuring multimorbidity from patients’, providers’ and health systems’ perspective were Patient Self-Reported Outcomes, Electronic Health Records and Ministry of Health administrative data, respectively. While existing literature highlights heterogeneity in prevalence estimates based on the type of data source used [54-57], there is no clear consensus on which data source to use for measuring multimorbidity. Hence, our findings will provide a good starting point in the local context to choose an appropriate data source based on different perspectives*.”

The change is reflected on pages – 32, lines 537 to 545.

Comment 11: The flowchart (Fig. 1) is very blurry. Perhaps it would be better not to insert it as a picture?

Response: Thank you for this comment. As per the PLOS ONE guidelines on figure, the permissible file format is either TIFF or EPS. Hence, conforming to this permissible file format and also to address this comment, we have revised the flowchart (Fig 1) to improve its quality.

The revised flowchart is uploaded along with the revised submission.

Comment 12: Tab. 5 extends over 3 pages. Is it possible to put this on one page?

Response: Thank you for this comment. While we understand reviewer’s perspective, it is not possible for us to edit the contents of a 3-page table into a 1-page table. Also, it will not be prudent to split this into multiple tables as it is important for us to present this information on the voting of the conditions in the list of conditions in one table for the readers’ understanding and from comprehensiveness perspective. Lastly, we would like to highlight here that we have conformed to the table formatting guidelines provided by PLOS ONE journal which generally does not restrict the size of tables.

We have also copied the guidance provided by PLOS ONE on size of tables here for reviewer’s kind reference (source: https://journals.plos.org/plosone/s/tables):

*Size.*

*Tables do not have strict width and height requirements. Do not split your table or otherwise try to make the table appear within the manuscript margins if it does not fit on one page. In Word, tables that run off of the manuscript page can be seen using Draft View. In the PDF version of the published article, very wide tables may be printed sideways, and long tables may span more than one page.*
