# Supplementary material for: Defining and measuring multimorbidity in primary care in Singapore: Results of an online Delphi study
Source: PLoS One. 2022 Dec 1;17(12):e0278559. doi: 10.1371/journal.pone.0278559 (PMC9714819; doi:10.1371/journal.pone.0278559)
Supplement: Attachment — Submitted filename: Response to Reviewers.docx [file pone.0278559.s007.docx]

Prof (Dr) Gerald Koh, MD, PhD

Clinical Director and Head, Future Primary Care (FPC)

MOH Office for Healthcare Transformation

1 Maritime Square, #12-10, Harbourfront Centre

Singapore 099253

[Gerald_koh@moht.com.sg](mailto:Gerald_koh@moht.com.sg)

Dr Elisa Panada, BSc, PhD

Associate Editor, PLOS ONE

March 11, 2022

Re: Resubmission of manuscript *Defining and measuring multimorbidity in primary care in Singapore: results of an online Delphi study,* Manuscript ID PONE-D-21-28758R1

Dear Dr Elisa Panada,

Greetings.

Thank you so much for seeing merit in our manuscript, entitled “Defining and measuring multimorbidity in primary care in Singapore: results of an online Delphi study,” and giving us the opportunity to revise it further. We really appreciate the feedback we have received.

We once again want to thank you and the reviewers for taking the time and effort to carefully read our work and provide us encouraging feedback, with constructive suggestions to work on. We believe that the manuscript is significantly improved after making the suggested amendments and we hope that it meets your expectations as well.

The revision has been developed in consultation with all co-authors, and each author has given approval to the final form of this revision. Changes made in the manuscript are marked using track changes in the “Revised Manuscript with Track Changes”. We have uploaded this track-changed copy along with a clean copy designated as “Manuscript”, and S1 Appendix. CREDES checklist (updated page numbers) for your kind perusal. We have no conflicts of interest to declare.

**Please find below the point-by-point response to all the comments by the Reviewers as required**.

Thank you for your kind consideration.

Sincerely,

Prof (Dr) Gerald Koh, MD, PhD

Clinical Director and Head, Future Primary Care (FPC), MOH Office for Healthcare Transformation

Professor, Saw Swee Hock School of Public Health, National University of Singapore

**POINT-BY-POINT RESPONSE TO COMMENTS BY REVIEWERS.**

Please take note that the page and line numbers mentioned in the responses below are referring to the revised version of the manuscript (marked copy with track-changes).

**Editor Comments.**

Comment: Thank you for submitting your manuscript to PLOS ONE. After careful consideration, we feel that it has merit but does not fully meet PLOS ONE’s publication criteria as it currently stands. Therefore, we invite you to submit a revised version of the manuscript that addresses the points raised during the review process.

The manuscript has been assessed by two reviewers, and their comments are appended below. One of the reviewers have raised major concerns regarding the analyses reported, the design of the study, the statistical analyses and the validity of the results reported. Please, could you carefully revise the manuscript to address the concerns raised?

Response: Thank you so much for taking the time to review our paper and providing us with this second round of comments/suggestions to improve our manuscript. We have tried to address the reviewer’s comments and made amendments as required. Hopefully, the revised manuscript will meet your standards.

**Reviewer 1:**

Comment 1: I appreciate the opportunity to review this paper again and appreciate the authors’ attempts to address review concerns. Unfortunately, the authors have made minimal changes to address the concerns brought forth by the reviewers. Many of the changes are surface level and not well-integrated into the paper and bring new issues to light. This paper remains potentially important however the importance of the study is in doubt as it is not well-justified in the Introduction or well-supported by the Methods and Results used to address the gap in the literature in a meaningful, accurate way.

Response: Thank you so much for seeing merit in the manuscript and acknowledging our attempts at the revisions to address first round of comments, which also resulted in Reviewer 2 accepting our revisions, which fully addressed the concerns raised by them. We really appreciate further constructive feedback given by Reviewer 1 and we have tried our best to address these in the revised manuscript for this second round of revisions. We believe that the incorporated revisions have further improved our manuscript and hopefully the revised manuscript will meet your standards as well.

Comment 2: The Methods and Results remain unclear. The authors also do not explain well how this new definition makes a helpful impact on the field. They merely explain how the definition is different than others but not why it is better, more accurate or more meaningful. The lack of clarity in the Methods, Results and Discussion make it difficult to determine the scientific validity of the work. Based on the manuscript as written, I do not see how readers would be able to use the authors’ new multimorbidity definition or why it would be chosen over an established definition. More complete rewrites are needed to address these methodological and scientific meaning concerns.

Response: Thank you for this comment. We would like to take this opportunity to highlight that our methodology was conceptualised, implemented and reported in accordance with the Recommendations for the Conducting and REporting of DElphi Studies (CREDES), which is the international criteria followed for such Delphi studies. Reviewer 2 had also noted this methodological rigour about our manuscript.

We would also like to thank reviewer 1 for providing us with extensive comments across our introduction (comments #2 to #9), methods (comment #10), results (comments #11 to #19) and discussion (comments #20 to #33) sections. We have addressed all these comments to the best of our ability and have made extensive and more complete re-writes for the introduction, methods, results and discussion sections. We believe that the revised manuscript is not only improved further but the comprehensive revisions incorporated also address all the points raised by the reviewer. These include clarity of the methods, results and discussion sections, relevance of our definition of multimorbidity from an epidemiological and clinical perspective and highlight the validity of our work in comparison to existing literature.. All these incorporated revisions have thus addressed the methodological and scientific meaning concerns raised by the reviewer.

We would like to highlight some of the main comments that we have addressed below, which we request the reviewer to refer to our responses for further details pertaining to this comment:

1. Comment 4, 7 and 9: related to introduction needing to demonstrate where there are meaningful differences in definitions, expansion of literature (including citations) to illustrate the lack of convergence or heterogeneity in existing literature and why Singapore-only, primary care-only definition is needed.
2. Comment 5: related to expansion of the methodological details of referenced 16 and explanation of why the range of prevalence is a clinical concern.
3. Comment 6: related to providing a justification for why this Delphi study was needed and limitations of the Fortin’s list in reference 16.
4. Comment 8: related to justification of our approach and how involving stakeholders under our adopted Delphi methodology would help develop interventions and tracking programs.
5. Comment 10: related to expanding on the work done in reference 16 and rationale by the authors to include pre-diabetes and physical disability, which will make the methods section clearer.
6. Comment 12: related to clarifying how the impact of chronic condition is used in the final revisions and that it is not a separate entity.
7. Comment 14: related to methodological clarification of how the panellists voted and, based on consensus, how cut-off of 3 or more conditions was finalised to illustrate how our recommendations are well-supported by our methodology and findings.
8. We have addressed/responded to the reviewer on why bipolar was a separate category as compared to anxiety and depression (comment #11), why gout was a separate category from OA/RA (comment #18) and why IBD was not included (comment #17).
9. Comment 24: related to asking for re-write of the discussion to better integrate the major additions incorporated in the discussion section to address all the comments from round 1 and round 2 given by reviewer 1.
10. Comment 26: related to explaining the science behind why our definition is more accurate or clinically relevant.
11. Comment 32: related to overall lack of depth and limited clarity on accuracy of definition of multimorbidity and why a new definition might better serve our audience clinically.

Comment 3: Introduction

-says that definitions are without much convergence and then lists 3 definitions that are the same: 2 or more conditions or health factors. The European GP Research Network could be considered different in that it names acute conditions or risk factors as being included along with a single chronic condition, rather than requiring 2 or more chronic conditions (which include risks factors), but these are not that different. More depth is needed here as well as more definitions that are cited later in the paper.

Response: Thank you for this comment. To address this, we have revised the text under the introduction section to highlight further the lack of convergence across these cited definitions. Also, the introduction is expanded to include more depth and information on lack of convergence across different aspects of multimorbidity definitions in existing literature (along with citation of supporting literature).

The change is reflected in the introduction section of the revised manuscript:

-	Page – 3, lines 58 to 64

-	Page – 4, lines 68 to 91

Comment 4: Introduction

-The intro needs to demonstrate where there are meaningful differences in definitions and why the authors’ new definition is needed. The Singaporean-only definition for primary care does not help with consensus across multiple countries or for comparison between studies outside PC in Singapore. So why is this definition needed? What does it give you? Why can’t a current definition that has already been developed and tested be used? Is there something unique in Singapore that requires its own definition? This must be justified more. It seems to me that per-country definitions will cause more fragmentation rather than clear comparisons between countries and regions, so you need to well-justify the need for country-specific multimorbidity definitions. (Especially as these definitions seem to be based on current prevalence patterns in the population that can change based on the population’s health and the doctor’s diagnostic patterns - so how often should these definitions be revisited?)

Response: Thank you so much for this comment. We would like to take this opportunity to elaborate that the intention of our study was to get consensus on the definition of multimorbidity in the Singapore primary care context in the absence of a universally recommended/accepted definition of multimorbidity. In such circumstances with abundance of literature and associated heterogeneity in the application of the same, a methodology such as the Delphi process is most suited to come to a consensus on which aspects of multimorbidity definition (from existing literature) are acceptable in a chosen context based on expert views. Otherwise it is not feasible to justify the arbitrary selection of 1 of the existing definitions and expect that it would be accepted unanimously within a particular setting. Our intention was not to internationally benchmark and recommend a definition that is universally accepted, rather our study had a defined scope of operationalising multimorbidity within our context, based on current existing literature and consensus voting by local experts in the field from an implementation perspective. Furthermore, by engaging the stakeholders (i.e., the expert Delphi panellists) and having a comprehensive representation from the primary care sector of Singapore would hopefully advance the development of interventions to improve outcomes for patients with multimorbidity within Singapore. While we agree that comparison across different countries of multimorbidity prevalence may be relevant from an epidemiological perspective (conditional on presence of a universally accepted definition to make such a comparison feasible, which currently is lacking), we should be equally understanding of the practical/clinical perspective of defining multimorbidity to facilitate quantification of the burden, resource allocation, development of interventions to improve the outcomes of patients with multimorbidity. The latter is the intention of our study as is specified in the aim of the study and throughout the manuscript. Hope this addresses the reviewer’s comment.

To further address this comment, we have expanded the introduction section to illustrate the detailed differences in the multimorbidity definitions across existing literature to explicitly highlight the heterogeneity and rationale for our study. Please refer to our response to comment 9 below for further details. Additionally, we request the reviewer to refer to our response to comment 7 below which addresses the point on why Singapore-only and primary care-only definition is needed.

Comment 5: Introduction

-The reference 16 describes multiple measures being compared but that methodological detail is not in this manuscript, nor is an explanation of why the range in prevalence found in #16 is a clinical concern (likely it is, but that should be supported in this manuscript.)

Response: Thank you for this comment. To address this, we have indicated in the introduction section of the revised manuscript the implication of variation in the prevalence. To summarise, this variation in the estimated prevalence is concerning as it impedes the quantification of multimorbidity burden, subsequent resource allocation and practice changes to improve the outcomes of patients with multimorbidity.

To further address this comment, we have added the methodological details used in reference 16 under the Methods section and discussion section of the revised manuscript. We have copied the added text here for the reviewer’s kind reference:

“To elaborate, the authors suggested a modified list of conditions for measuring multimorbidity on the basis of relevance in primary care in Singapore, conditions with high burden and number of chronic conditions in a list. The relevance in primary care setting of Singapore was determined by “consensus reached after iterative discussions between clinicians, research team members and reference to statistics from the MOH and local primary care initiatives”. [19] A condition was considered to have high burden if the standardised prevalence ratio was 1% and above in the primary care setting in Singapore. The authors considered lists comprising of 12 conditions or more as per the previously recommended appropriate threshold. [10] Additionally, ‘pre-diabetes’ and ‘physical disability’ were added to the list to increase the comprehensiveness. Pre-diabetes was added to align with the local context as Singapore government has placed significant emphasis on management of individuals with diabetes and pre-diabetes. [45] Additionally, pre-diabetes is part of the Chronic Disease Management Program (CDMP) in Singapore, which was introduced in Singapore in 2006 to provide guidance on management of commonly occurring chronic conditions in primary care and reducing out-of-pocket payment for such conditions by introducing financial measures. [46] Physical disability was included as it comprised of conditions like hearing loss which are relevant in the Singapore context due to aging population as well as considering the multi-dimensional impact of such conditions on the patients. [19] While this modified list of conditions was a good starting point for seeking feedback from the Delphi panellists, it is important to note that it could not be directly adopted for measuring multimorbidity since it was recommended based on relative performance in comparison with the other locally used definitions of multimorbidity in Singapore. Secondly, the validity of these definitions could not be assessed completely in the absence of sufficient methodological details. Thirdly, contextual relevance and acceptance by local stakeholders were not known. [19] Lastly, Singapore has a hybrid healthcare system comprising of both public and private providers. [47] Within the public primary care, the primary care clinics are organised into three clusters to better meet the needs of the patients. Since the above study was based in one of these three public primary care clusters with no representation from private providers of primary care, it’s findings may not be reflective of the whole primary care in Singapore. Hence, there was a need to seek feedback from stakeholders across both public (including all three clusters) and private settings to derive a consensus-based definition of multimorbidity.”

“From an epidemiological perspective, while having a higher cut-off or threshold for measuring multimorbidity is associated with a lower prevalence, having a longer list of conditions is associated with a higher prevalence. Although looking at such individual dimensions for defining multimorbidity across different studies may present conflicting impact on prevalence estimation, it is important to understand that the definition of multimorbidity is multi-dimensional and hence, it is not plausible to exactly know the impact of a recommended definition on prevalence of multimorbidity when compared with others. Moreover, it is more meaningful to compare the clinical relevance of such definitions along with understanding the accuracy of developing such definitions. From a clinical perspective, our definition is more meaningful compared to local literature since with a higher cut-off, it would result in identification of patients with multimorbidity with higher care needs, who would benefit from the holistic management by a family physician in a primary care setting. It is not feasible to assess the accuracy of the local existing studies as none of them explained in detail the science behind developing their definition. In contrast, our recommended definition of multimorbidity, including the list of 23 conditions, was a result of consensus-voting by a group of Delphi panellists, who had both expertise in the field of multimorbidity and practical insights into managing patients with multiple chronic conditions in a primary care setting. Therefore, our multimorbidity definition and list of conditions are potentially more suited for defining and measuring multimorbidity within the Singapore primary care setting. n the absence of a universally recommended definition of multimorbidity with simultaneous presence of heterogenous ways of defining multimorbidity and lack of consensus, our chosen Delphi methodology is one of the most appropriate methods to gain consensus. Moreover, the conduct and reporting of our Delphi study was done in accordance with CREDES [37], which illustrates the robustness of our study and the validity of our findings. Throughout the study, we upheld the core tenets of the Delphi process, i.e., maintaining quasi-anonymity, practising iteration and controlled feedback, responses of the group being analysed in a statistical manner and compared with pre-defined consensus thresholds and sharing of results with Delphi panellists after each round. All of the above led to the final recommendation of a consensus-derived, context-based, clinically relevant definition of multimorbidity, which was rooted in sound methodology and well-supported by our study findings.”

The change is reflected in the revised manuscript:

-	Page – 5, lines 93 to 96

-	Pages 11 – 12, lines 248 to 280

-	Pages 43 – 45, lines 724 to 753

Comment 6: Introduction

-If #16 found that Fortin’s list is the best and prediabetes and physical diabetes should be added, why is this Delphi study needed? What does the study add to what was found in #16? That justification would help.

Response: Thank you for this comment. To address this, we have expanded the description of the modified list of conditions recommended in reference #16 to explain why our Delphi study was needed. To summarise, in reference #16, Fortin’s list was recommended based on relative performance in comparison to the local definitions of multimorbidity previously used in Singapore. Due to lack of sufficient details related to the methodology of development of these definitions, their validity cannot be assessed. Additionally, the contextual relevance and acceptance by local stakeholders was unknown. Lastly, this study was based only in one part of the public primary care of Singapore without any representation of the private primary care. Hence, there was a need to seek feedback from stakeholders across both public (including all three clusters) and private primary care providers to derive a consensus-based definition of multimorbidity. Our study has addressed this gap.

We have copied the added text here for reviewer’s kind reference:

“While this modified list of conditions was a good starting point for seeking feedback from the Delphi panellists, it is important to note that it could not be directly adopted for measuring multimorbidity since it was recommended based on relative performance in comparison with the other locally used definitions of multimorbidity in Singapore. Secondly, the validity of these definitions could not be assessed completely in the absence of sufficient methodological details. Thirdly, contextual relevance and acceptance by local stakeholders were not known. [19] Lastly, Singapore has a hybrid healthcare system comprising of both public and private providers. [47] Within the public primary care, the primary care clinics are organised into three clusters to better meet the needs of the patients. Since the above study was based in one of these three public primary care clusters with no representation from private providers of primary care, its findings may not be reflective of the whole primary care in Singapore. Hence, there was a need to seek feedback from stakeholders across both public (including all three clusters) and private settings to derive a consensus-based definition of multimorbidity.”

The change is reflected in the revised manuscript, page – 12, lines 267 to 281.

Comment 7: Introduction:

-the authors revisions to justify why PC are incomplete. More clear justification is needed on why a Singapore-only, PC-only definition is needed compared to using a current definition. Why are current definitions more faulty for Singaporean PC than for other populations? Multimorbidity definitions do lack some consensus and do have their faults, but why does there need to be on for Singapore primary care particularly? Do the authors argue that each country should have their own PC definition?

Response: Thank you so much for this comment. We would like to take this opportunity to clarify that we have not indicated in our manuscript that the current definitions are faulty or inaccurate. Instead the main issue is that there is no universally accepted/recommended definition of multimorbidity that different countries are currently using, which is what we have described in our introduction section. To address this comment, we have further expanded the rationale (in the revised manuscript) for why we focussed our research efforts to develop a consensus-derived definition of multimorbidity within the Singapore primary care setting.

We have copied the revised text here for the reviewer’s kind reference:

“It is important to describe multimorbidity within a specified setting as the prevalence of multimorbidity has been reported to vary across different settings. [10] It has also been reported that the reliability of measures of multimorbidity used in one setting may not translate directly into another setting. [21] Moreover, previous studies exploring multimorbidity have also adopted such a setting-specific approach. [17, 21] With this setting-specific approach, primary care is aptly suited to provide patient-centred holistic care for patients with multiple chronic conditions based on the tenets of continuity, coordination and comprehensiveness. [22] Almost 8 in 10 consultations in a primary care setting may involve a patient with multiple chronic conditions. [23] From a management perspective, primary care setting is aptly suited to manage multimorbidity with the ability to adopt the paradigm of “goal orientation” as opposed to “disease orientation”. Family physicians can help patients to define goals and integrate such goals in their clinical decision-making for multimorbidity management. [24] Additionally, it is established that management of multiple chronic diseases by interventions within primary care setting are more cost-effective as compared to other care settings. [25] Hence, for the current study, we focussed on developing a consensus-derived definition of multimorbidity within the primary care setting.

With current lack of a universally recommended approach to defining and measuring multimorbidity and resultant heterogeneity in existing literature, it is important to adapt the existing multimorbidity related concepts based on contextual relevance and consensus opinion of experts. It has been reported that “locally-generated research evidence” is more likely to be translated into practice as it is highly valued by the policy makers and practitioners and perceived to be contextually relevant. [26] Hence, the current study is based within Singapore’s primary care setting, which is also aligned with the intention of translating the research findings from this study into practice changes to improve management of multimorbidity and related outcomes.”

The change is reflected in the introduction section of the revised manuscript, pages 5 – 6, lines 105 to 129.

Comment 8: -How will having this be stakeholder driven will help develop interventions and tracking programs. The ideas are here but need clarity and specifics from the literature.

Response: Thank you for this query. Stakeholder engagement is important to bridge the gap between research evidence and clinical practice and this principle is applicable for the current work scope as well. Involving stakeholders will potentially increase ownership and adoption of the consensus-derived definition of multimorbidity. It has been reported in literature that research efforts aimed at improving the outcomes of intended end-users at scale have the greatest impact when the knowledge generation and application in practice setting are shared between researchers and stakeholders or decision-makers. This shared responsibility is achieved by co-production of knowledge between researchers and relevant stakeholders. Hence, involvement of local stakeholders in our setting for development of consensus-derived definition of multimorbidity would facilitate the adoption of this definition, development of interventions and improve management of patients with multimorbidity. To address this comment, we have included this text in the introduction section of the revised manuscript with the relevant citations from literature.

The change is reflected in the introduction section of the revised manuscript, page – 6, lines 132 to 140.

Comment 9: -“there still remains heterogeneity in both the overall methodology and the list of chronic conditions considered, highlighting the need to develop a new, consensus-derived definition of multimorbidity within the primary care setting in Singapore.” This needs specific justification and citations. Is this referring to the work in #16? Those details need to be above (don’t make the reader go to the cited paper in order to understand your justification) and then restating in summary here.

Response: Thank you for this query. To address this, we have expanded the text under the introduction section of the revised manuscript to provide details of areas of heterogeneity in existing literature across different dimensions of defining multimorbidity (i.e., 1) definition of chronic conditions, 2) list of conditions considered, 3) cut-off for the number of conditions used to define multimorbidity and 4) data sources to confirm conditions). This detailed explanation of heterogeneity is applicable to Singapore context as well as indicated in our manuscript and proves the required justification.

We have copied the added text here for the reviewer’s kind reference:

“One review comparing studies on multimorbidity prevalence reported lack of consistency in the adopted definition of a chronic condition in included studies. [10] Another review of literature concluded that a chronic condition is a multi-faceted concept, comprising of characteristics like duration, onset, prognosis, pattern, aetiology etc. and reported the variations in existing literature across these characteristics without any consensus. For example, use of different time intervals to describe duration with the commonly reported intervals being 3, 6 and 12 months. [16] While one systematic review reported that the list of conditions in included publications had 5 to 335 conditions. [17], another summarised the number of conditions across 566 studies to range from 2 – 10 (18% of studies), 11 – 20 (40% of studies), 21 – 30 (12% of studies), 31 – 40 (6% of studies), 41 – 50 (3% of studies) and more than 50 (4% of studies), highlighting heterogeneity in the reported list of conditions. [18] One systematic review included 39 studies across 12 countries summarising prevalence and patterns of multimorbidity in primary care reported 25 studies using a cut-off of 2 or more conditions, while 5 studies reported a cut-off of 3 or more conditions. [17] Another systematic review reported studies adopting different cut-offs of 2 or more, 3 or more and 4 or more conditions. [10] A recent systematic review examining the definition and measurement of multimorbidity in international literature reported 206 studies not specifying any cut-off, 268 studies specifying a cut-off of 2 or more conditions and 28 studies specifying a cut-off of 3 or more conditions. [18] This illustrates the heterogeneity in adoption of a cut-off for the number of conditions used to define multimorbidity without any clear consensus or recommendation for one specific cut-off. Data sources to measure multimorbidity were also reported to vary between patient self-reports (55% of studies) or medical records and administrative databases (42% of studies) or a combination of the two (1% of studies) illustrating the heterogeneity in existing literature. [18]”

The change is reflected in the introduction section of the revised manuscript, page 4, lines 68 to 87.

Comment 10: Methods:

-“The list proposed 192 by Fortin et al. [37] was modified to make it suitable for use in primary care setting in Singapore 193 with inclusion of pre-diabetes under the chronic condition of ‘diabetes’ and also inclusion of 194 ‘physical disability’ in the modified list.” This section needs to also explain that the work in 16 found that this definition had the most accurate prevalence (if true) and how the authors concluded that prediabetes and physical disability were needed.

Response: Thank you for this query. To address this, we have expanded this section to explain the work done in reference 16 and the rationale by the authors for including pre-diabetes and physical disability. We have copied the text here for reviewer’s kind reference:

“To elaborate, the authors suggested a modified list of conditions for measuring multimorbidity on the basis of relevance in primary care in Singapore, conditions with high burden and number of chronic conditions in a list. The relevance in primary care setting of Singapore was determined by “consensus reached after iterative discussions between clinicians, research team members and reference to statistics from the MOH and local primary care initiatives”. [19] A condition was considered to have high burden if the standardised prevalence ratio was 1% and above in the primary care setting in Singapore. The authors considered lists comprising of 12 conditions or more as per the previously recommended appropriate threshold. [10] Additionally, ‘pre-diabetes’ and ‘physical disability’ were added to the list to increase the comprehensiveness. Pre-diabetes was added to align with the local context as the Singapore government has placed significant emphasis on management of individuals with diabetes and pre-diabetes. [45] Additionally, pre-diabetes is part of the Chronic Disease Management Program (CDMP) in Singapore, which was introduced in Singapore in 2006 to provide guidance on management of commonly occurring chronic conditions in primary care and reducing out-of-pocket payment for such conditions by introducing financial measures. [46] Physical disability was included as it comprised of conditions like hearing loss which are relevant in the Singapore context due to aging population as well as considering the multi-dimensional impact of such conditions on the patients. [19]”

The change is reflected in the methods section of the revised manuscript, pages 11 – 12, lines – 250 to 267

Comment 11: Results

-the authors did not explain why bipolar disorder was grouped separately from anxiety and depression. They seem to have made that choice separately from what the Delphi round participants suggested. The authors seemed to have started with the idea of Fortin + prediabetes and physical disability and ended there. It is not clear if they used any data from Delphi to adjust their definition.

Response: We decided to keep bipolar disorder separately from anxiety and depression category since the Delphi panellists did not recommend to subsume bipolar disorder under the latter category. This was to align with the feedback from Delphi panellists and ground our findings/recommendations within our methodology. Based on feedback from Delphi panellists in Round 1, we included new additions into the original list. Subsequently, we asked the Delphi panellists to vote on inclusion/exclusion of conditions included in the list in Rounds 2 and 3. Open feedback was also sought from the panellists for each condition for comprehensiveness. None of the panellists suggested to include bipolar disorder under the category of anxiety and depression.

Additionally, to make the changes in the list of conditions throughout the Delphi process explicit, we have also added Table 6, which explicitly illustrates the new additions after Round 1 and outcome of voting from Round 2 and 3.

The change is reflected in Table 6 of the revised manuscript.

Comment 12: Results

-how was the impact of the chronic condition (Table 3) used in the final definition of multimorbidity? That seems to be included as a separate entity and not incorporated.

Response: Thank you for this query. To clarify, the impact of chronic condition is one of the parameters to describe ‘chronic condition’, which in turn is part of the definition of multimorbidity. We would like to highlight that we had included in our original manuscript, under the Results section, the final consensus-derived definition of chronic condition after 3 Delphi rounds to be inclusive of the impact of the chronic condition.

We have copied this text here for the reviewer’s kind reference:

“Therefore, the consensus derived criteria for a condition to be defined as chronic based on finalised four components is as follows: 1) it should last for six months or more (duration); 2) it should be recurrent or have a persistent course (recurrent or persistent course); 3) it should impact the patient in one or more of the following dimensions: limitations in activities of daily living or instrumental activities of daily living or physical disability, mortality and psychological impairment (impact on the patient) and 4) it should require long-term follow-up (management of patient).”

Additionally, to further emphasise the incorporation of the impact of chronic condition as one of the criteria for defining a condition as chronic and in turn part of the definition of multimorbidity, we have included the final consensus-derived definition of multimorbidity under the Discussion section of the revised manuscript (inclusive of the definition of chronic condition).

The change is reflected in the discussion section of the revised manuscript, page – 33, lines – 485 to 493.

Comment 13: Results

-Why does Table 3 have empty columns for Round 3? That is odd. These should be removed and a comment added to the legend that these items were not considered din round 3. Otherwise, fill in the table for Round 3 with “not considered.”

Response: Thank you for this query. To clarify, Table 3 has empty columns for Round 3 since the criteria for consensus was met in Round 2 and none of the items in Table 3 were taken into Round 3. This information was originally provided in the footnote of the table. The columns related to Delphi Round 3 were retained to maintain consistency across different tables summarising the findings from different Delphi Rounds. To further clarify this point, we have indicated in the revised manuscript “Not Considered” for Round 3 as kindly suggested by this reviewer.

The change is reflected in Table 3 of the revised manuscript.

Comment 14: Results

-For the cut off of conditions, how many panelists agreed to 2 or more conditions (the current most common count per your introduction). How many to 1 condition plus an acute condition or other risk factor? (Per the Euro GP Research Network)? It seems the authors are only presenting the findings that support their final conclusions but the readers will want to see the other relevant data.

Response: Thank you for this query. To address this query, we would take this opportunity to clarify that we have not endorsed any cut-off as the most common in our Introduction section of the manuscript. Rather we have mentioned that there is currently no consensus in terms of what cut-off to be used to define multimorbidity.

We have substantiated our results with data as much as possible throughout our manuscript. For this specific cut-off related comment, we would like to highlight that we had included in the previously submitted manuscript under the Delphi Round 1 (which was qualitative) findings (page – 12), the number of respondents who reported the most frequent cut-offs (i.e., 13/34 panellists indicated 3 or more; closely followed by 2 or more conditions) for defining multimorbidity. We have copied the text here for reviewer’s kind reference as well:

“The most commonly reported cut-off was three conditions and above, which was reported by 13 of the 34 panellists. The second most commonly reported cut-off was two conditions and above.”

To further clarify and address reviewer’s comment, we have expanded the text under the findings of Delphi Round 1 (i.e., qualitative) in the revised manuscript to provide number of respondents who suggested 4 or more and 5 or more conditions as a cut-off. Please kindly note that participants suggested 2 or more, 3 or more, 4 or more and 5 or more as the cut-offs for defining multimorbidity in this qualitative round 1.

We have copied here the revised text for the reviewer’s kind reference:

“The most commonly reported cut-off was three conditions and above, which was reported by 13 of the 34 panellists. The second most commonly reported cut-off was two conditions and above (8 out of 34 panellists). Other cut-offs suggested by panellists included five or more conditions (5 out of 34 panellists) and four conditions and above (3 out of 34 panellists).”

Comment 15: Results

-The conditions in S3 and S4 need to be listed in the main manuscript, not buried in a survey in the appendix.

Response: Thank you for this comment. The list of conditions in the survey questions in S3 and S4 corresponding to Delphi Round 2 and 3 are already provided in Table 5 of the manuscript along with details of the voting for each round and whether consensus was achieved or not. Please refer to Table 5 for more details.

Comment 16: Results

-The results from each ound are unnecessarily wordy and hard to follow. A terse re-write would benefit the reader.

Response: Thank you for this suggestion. To address this, we have revised the results section to try to make it shorter along with balancing the accurate and sufficient reporting of our findings to support our recommendations .

The changes are reflected throughout the results section of the revised manuscript.

Comment 17: Results

- The authors did not address when IBD is left out.

Response: Thank you for highlighting this. To clarify, IBD was not included in the finalised list of conditions since it was not present in the original list of conditions which was presented to the panellists in Delphi Round 1 (which was adapted from list of conditions proposed by Fortin et al., based on relevance in primary care), only 1 panellist suggested to consider adding IBD under the colon problems category, IBD itself is rare in Singapore population (as compared to western population, where the prevalence is high) and within the Singapore healthcare system, patients with IBD are generally seen in specialist care settings and not in the primary care setting.

We have added this information in the revised manuscript to address this comment.

The change is reflected in the revised manuscript, page – 17, lines 370 to 373.

Comment 18: Results

-why is gout separate from OA or RA?

Response: Thank you for this query. We decided to keep gout as a separate category from OA/RA since the Delphi panellists did not recommend to subsume gout under the latter category. Moreover, gout is predominantly a metabolic condition unlike OA and RA.

Comment 19: Results

-It is very hard to follow which conditions were provided to the panelists in Round 1 and which they suggested themselves, then how these were presented in Round 2 and Round 3 to get to your consensus. It is also hard to see how the list developed through this work compares to what fortis et al (or others) suggest. A clear flow diagram and table would help. Without clarity in these methods and results, we cannot see the scientific validity of the work.

Response: Thank you for this suggestion. To address this, we have included a new table in the revised manuscript to present the consolidated findings for the list of conditions across Delphi rounds 1 to 3 beginning with the original list used.

The change is reflected in the revised manuscript, Table 6.

Comment 20: Discussion

-The first paragraph should provide your concise definition (your study findings) and possibly suggested use of the definition.

Response: Thank you so much for this suggestion. We have included the concise definition of multimorbidity and suggested use of the definition.

The change is reflected in the revised manuscript, page – 34, lines 485 to 493.

Comment 21: Discussion

-The discussion next seems to present new qualitative findings and methods. All results and methods should be in their sections. The discussion should serve to place these findings in the current literature and give implications.

Response: Thank you for highlighting this. To address this comment, we have removed this text from the discussion of the revised manuscript.

Comment 22: Discussion

-the focus in the discussion on 2 vs 3 as a cut off needs to be set up in the intro and be well-supported by your research methodology and results. That is ignored in the paper.

Response: Thank you for this comment. To address this, we have included new text in the introduction section of the revised manuscript describing the adoption of 2 vs 3 as cut-off for defining multimorbidity in the current literature to highlight the heterogeneity and also lack of consensus/recommendation of adoption of 1 cut-off over other.

Additionally, we would like to re-iterate that our consensus-derived recommendation of cut-off of 3 or more conditions is well-supported by our research methodology and results. We would like to refer the reviewer to our response to comment 14 above which is related to this comment 22. To elaborate further, we have substantiated our results with data as much as possible throughout our manuscript. For this specific cut-off related comment, we would like to highlight that we had included in the previously submitted manuscript under the Delphi Round 1 (which was qualitative) findings (page – 12), the number of respondents who reported the most frequent cut-offs (i.e., 13/34 panellists indicated 3 or more; closely followed by 2 or more conditions) for defining multimorbidity. We have copied the text here for the reviewer’s kind reference as well:

“The most commonly reported cut-off was three conditions and above, which was reported by 13 of the 34 panellists. The second most commonly reported cut-off was two conditions and above.”

To further clarify and address the reviewer’s comment, we have expanded the text under the findings of Delphi Round 1 (i.e., qualitative) in the revised manuscript to provide number of respondents who suggested 4 or more and 5 or more conditions as a cut-off. Please kindly note that participants suggested 2 or more, 3 or more, 4 or more and 5 or more as the cut-offs for defining multimorbidity in this qualitative round 1.

Lastly, we have also revised our discussion section (where we have included discussion on 2 vs 3 cut-offs) by clearly stating our results to highlight that the focus in the discussion on 2 vs 3 as a cut off is well-supported by our research methodology and results.

The change is reflected in the revised manuscript:

-	Introduction section, page 4, lines 79 to 88

-	Discussion section, page – 36, lines 538 to 543

Comment 23: Discussion

-What does this mean:” The inclusion of acute conditions for defining multimorbidity may be more relevant in developing settings where the prevalence of such conditions is higher as compared to a developed setting like Singapore. [47]” Europe is a developed setting. Is the European GP Research Network actually in developing settings?

Response: Thank you for this query. To address this, we have amended this text in the revised manuscript to specify that the exclusion of acute conditions is aligned with the Singaporean context of a developed country, with a rapidly aging population and a high prevalence of chronic diseases. Also, the epidemiology of multimorbidity in such a context is mainly characterised by chronic diseases, which accumulate with increasing age.

The change is reflected in the revised manuscript:

-	Under the discussion section, page – 38, lines 585 to 592.

Comment 24: Discussion

-the authors have made helpful additions to the discussion based on reviewer feedback but this is not well integrated into the discussion resulting in a disjointed discussion. The discussion should be re-written to fully incorporate the comparison with current literature and implications on the authors proposed new definition.

Response: Thank you so much for this comment. We have re-written the discussion section in the revised manuscript to fully incorporate the comparison with current literature, fully integrate the reviewer feedback into the revised discussion section in a coherent manner and incorporate the implications of our proposed new definition throughout the discussion. The re-written discussion section is organised as follows:

- Overall summary of the findings with description of the recommended definition along with implications.
- Comparison with international literature on (1) definition of chronic condition, (2) discussion on cut-off of 2 vs 3, (3) discussion on number of conditions included in the finalised list, (4) discussion on the types of conditions included/excluded in the finalised list with inclusion of chronic pain, (5) categorising the conditions in the final list into the multimorbidity framework of diseases, risk factors and symptoms.
- Comparison with local literature on cut-off of 2 vs 3, number of conditions included in the finalised list, types of conditions included in the finalised list.
- Summary of implications of the recommended definition of multimorbidity (with compared with other literature) from an epidemiological perspective, clinical perspective and accuracy perspective.
- Discussion on burden of multimorbidity being measured from different perspectives.
- Strengths and Limitations.
- Practical implications from our work.

The change is reflected throughout the discussion section of the revised manuscript.

Comment 25: Discussion

-“Comparing our finalised list of conditions with existing local literature, 4four [5, 8, 26, 468 50] out of the 5five studies from Singapore had the total number of conditions in their list lower than the 23 conditions included in our finalised list. Hence, the multimorbidity prevalence estimates generated by our list are expected to be higher as compared to most of previous studies from Singapore. When comparing the type of conditions included across these studies, our finalised list of conditions included all conditions from three of the above four studies. [8, 25, 26, 50] For the study by Quah and colleagues, we included all but one condition of gastrointestinal diseases based on feedback from the Delphi panellists. [5]” More is needed here. What does it mean if your multimorbidity estimates are higher? Of course they will be higher, more conditions will be included, but is that correct? Are your conditions more accurate because they were brought in by the Delphi methods? What were the definitions used in the other papers and why are they inadequate?

Response: Thank you for this query. We have expanded on this section in the revised manuscript to elaborate on why the definitions used in the other local papers were inadequate and also highlighted the differences between our recommended definition and the definitions used in these previous local studies. Additionally, since our list of 23 conditions was a result of consensus-voting by a group of Delphi panellists, who had both expertise in the field of multimorbidity and practical insights into managing patients with multiple chronic conditions in a primary care setting, our list is potentially more suited for defining and measuring multimorbidity within the Singapore primary care setting as compared these previous studies which often did not provide the methodology for their adopted definition of multimorbidity.

The change is reflected in the revised manuscript, page 43-45, lines 714 to 753.

Comment 26: Discussion

-In the Discussion, it is not enough to simply list that you have a higher cut off and more conditions, so you will have both a higher threshold to identify multi-morbidity (3+) and a lower one (more conditions). You needed to explain the science behind why your definition is more accurate or clinically relevant, so it is more correct than the previous definitions. Being different is not enough. The readers want to hear how your scientific method was better, resulting in a more accurate and more useful definition.

Response: Thank you so much for this suggestion. To address this, we have expanded the discussion section to explain the science behind why our definition is more accurate or clinically relevant when compared to the previous definitions. Additionally, we have provided information on how our scientific method was better, resulting in a more clinically relevant and more useful definition within the context of primary care in Singapore.

We have copied the added text here for reviewer’s kind reference:

“It is not feasible to assess the accuracy of the local existing studies as none of them explained in detail the science behind developing their definition. In contrast, our recommended definition of multimorbidity, including the list of 23 conditions, was a result of consensus-voting by a group of Delphi panellists, who had both expertise in the field of multimorbidity and practical insights into managing patients with multiple chronic conditions in a primary care setting. Therefore, our multimorbidity definition and list of conditions are potentially more suited for defining and measuring multimorbidity within the Singapore primary care setting. In the absence of a universally recommended definition of multimorbidity with simultaneous presence of heterogenous ways of defining multimorbidity and lack of consensus, our chosen Delphi methodology is one of the most appropriate methods to gain consensus. Moreover, the conduct and reporting of our Delphi study was done in accordance with CREDES [37], which illustrates the robustness of our study and the validity of our findings. Throughout the study, we upheld the core tenets of the Delphi process, i.e., maintaining quasi-anonymity, practising iteration and controlled feedback, responses of the group being analysed in a statistical manner and compared with pre-defined consensus thresholds and sharing of results with Delphi panellists after each round. All of the above led to the final recommendation of a consensus-derived, context-based, clinically relevant definition of multimorbidity, which was rooted in sound methodology and well-supported by our study findings.”

The change is reflected in the revised manuscript, discussion section, page 44-45, lines 735 to 753.

Comment 27: Discussion

-“Willadsen et al. [17] summariszed …..” You need to comment on if your Delphi panel thought risk factors and symptoms were needed. Also, state who decided to call “chronic pain” a symptom and not a condition (state if the 3 lists you give are from #17 or another source.)

Response: Thank you for this query, to address this we have explicitly stated in the revised text that while the conditions suggested or voted for by the Delphi panellists in the current study span across diseases, risk factors and symptoms, they did not explicitly categorise these conditions into diseases, risk factors and symptoms. However, comparing our finalised list of 23 conditions with the most frequently reported diseases, risk factors and symptoms by Willadsen and colleagues, we report that our finalised list comprises diseases like diabetes, cardiovascular diseases, stroke etc., risk factors like hypertension, hyperlipidaemia, obesity etc., and symptoms like chronic pain, hearing loss, incontinence etc.

The change is reflected in the revised manuscript, page 40, lines 635 to 642.

Comment 28: Discussion

-Again in the above paragraph you state that your measure will identify more patients with multimorbidity but do not mention if that is more accurate or what it means for clinical care.

Response: Thank you for highlighting this. To address this comment, we have expanded the above paragraph to describe how inclusion of diseases, risk factors and symptoms would result in a clinically more meaningful definition as compared to inclusion of only diseases.

We have copied the included text here for reviewer’s kind reference:

“From a clinical perspective, inclusion of diseases, risk factors and symptoms in our finalised list for defining multimorbidity is clinically more meaningful than inclusion of just diseases since the latter will result in exclusion of the patients’ views which are mainly captured by risk factors and symptoms. Capturing the patients’ views in turn will result in more holistic management of patients with multimorbidity. From the physicians’ viewpoint, including risk factors enables awareness and prevention of future illness which may be associated with higher disease burden and lower quality of life for the patient. Finally, inclusion of symptoms is important to quantify multimorbidity as they not only enable the physician to make a clinical diagnosis, but also represent patients’ self-rated health.”

The change is reflected in the revised manuscript, page 40, lines 645 to 653.

Comment 29: Discussion

-In the discussion, the definition of chronic condition and rationale around stomach problems seem to contradict with including symptoms (pain) or risk factors (asymptomatic hypertension). This needs to be more clearly explained.

Response: Thank you for this query. To address this query, we would like to elaborate how symptom like chronic pain and risk factor like hypertension is aligned with our definition of chronic condition.

For chronic pain, the Delphi panellists provided qualitative feedback that this should be included when it is long-standing and requires the patient to modify his/her lifestyle and may need regular review and long-term treatment (aligned with criteria (1) of a condition being defined as chronic, i.e., lasting for 6 months or more, criteria (2) should have a persistent course and criteria (4) it should require long-term follow-up). They further shared that chronic pain contributes to morbidity, impacts patient’s quality of life, affects physical function as well as mental health and limits socio-occupational functioning (aligned with criteria (3), i.e., impacts patient in 1 or more of the following dimensions: limitations in ADL/IADL or physical disability, mortality etc.). We have included this qualitative feedback in the revised manuscript under the results section to further address this comment.

Risk factors like hypertension are also aligned with our definition of chronic condition since these are long-term conditions, which often require long-term follow-up and management in a care setting and impact the patient in one of the dimensions quoted in criteria (3). Additionally, though not within the scope of our manuscript, but there is evidence of hypertension being associated with poor quality of life. [1]

Reference [1]: Trevisol DJ, Moreira LB, Kerkhoff A, Fuchs SC, Fuchs FD. Health-related quality of life and hypertension: a systematic review and meta-analysis of observational studies. Journal of hypertension. 2011 Feb 1;29(2):179-88.

For the rationale given for stomach problems, as stated in the manuscript, it is based on the qualitative feedback provided by the Delphi panellists. Based on qualitative feedback received, the panellists (N=17) perceived stomach problems to be benign, and not have a significant impact on the patient which is generally aligned with the definition of chronic condition derived from this current Delphi study and does not contradict the definition. Additionally, we would like to highlight that the exclusion of stomach problems was based primarily on objective, quantitative voting by the Delphi panellists in Round 2 and 3 and qualitative feedback from the panellists is complimentary and not a stand-alone evidence/justification of inclusion/exclusion of conditions like stomach problems.

The change is reflected in the revised manuscript, page – 17, lines 374 to 379.

Comment 30: Discussion

-“However, currently, there are no standardised, recommended codes for 531 documenting chronic pain in the public primary care coding system in Singapore. While one 532 previous study reported being unable to map available ICD-10 codes to the category of ‘chronic 533 musculoskeletal condition causing pain or limitation’, [16] only one out of the remaining 534 previous studies measuring multimorbidity in Singapore included chronic pain in their list of 535 conditions.” How is there no way to diagnose chronic pain in Singapore? There are numerous ICD-10 codes for chronic pain. This needs more explanation. Also the “only one of the remaining studies” needs a citation. This sentence is hard to follow.

Response: Thank you for this comment. While there may be codes in the ICD-10 system for coding chronic pain, there is no universally accepted ICD-10 codes which can be used uniformly by family physicians to code chronic pain in the primary care system in Singapore. This is also reported in literature as none of the major international diagnostic coding systems (including ICD-10) include specific codes for chronic pain. Additionally, it has been reported that this unavailability of systematic coding for chronic pain may result in inconsistent coding using ill-defined symptom based codes (Smith BH, 2019). Hence, there is a need to engage the relevant stakeholders in the primary care sector in Singapore to derive mutually agreed upon coding principles for chronic pain using this ICD-10 coding system. To address this query, we have amended the text in the revised manuscript to incorporate this explanation. Additionally, as required, we have also included the citation for “only one of the remaining studies” in the revised manuscript

Reference: Smith BH, Fors EA, Korwisi B, Barke A, Cameron P, Colvin L, Richardson C, Rief W, Treede RD. The IASP classification of chronic pain for ICD-11: applicability in primary care. Pain. 2019 Jan 1;160(1):83-7.

The change is reflected in the revised manuscript, page – 39, lines 615 to 621.

Comment 31: Discussion

-“Hence, our findings will provide a good starting point in the local context to 547 choose an appropriate data source based on different perspectives.” How do you suggest suing these 3 different data sources? Choose one? Add them together? Average?

Response: Thank you so much for this comment. To address this, we have included additional explanation in the revised manuscript of how/in what contexts, researchers may choose a particular data source. To align with the recommendations given by the Delphi panellists and ensuring our practical implications are well-supported by our research work, our explanation is based on the perspective (patients’, providers’ and health systems’ perspective) of studying multimorbidity and we did not suggest any summation measures like average.

We have copied the added text below for the reviewer’s kind reference:

“For example, if researchers are studying multimorbidity from patients’ perspective (e.g., impact on multimorbidity on quality of life, self-rated health or treatment burden etc.), they may want to include Patient Self-Reported Outcomes as the data source to measure multimorbidity. Similarly, if researchers are studying multimorbidity from provider’s perspective (e.g., association of multimorbidity with consult time), they may want to include Electronic Health Records as the data source to measure multimorbidity. Lastly, if researchers are studying multimorbidity from health systems’ perspective (e.g., quantifying the healthcare utilization associated with multimorbidity), they may want to include Ministry of Health administrative data as the data source to measure multimorbidity.”

The change is reflected in the revised manuscript, page – 48, lines 828 to 837.

Comment 32: Discussion

-The Discussion overall lacks depth and seems to not understand why an accurate definition of multimorbidity is needed or what it means. The authors contradict themselves, supporting the benefits of a stricter definition of multimorbidity with 3+conditions to have an underestimate of multimorbiity such that those identified are the sickest. But then the authors say that they include more conditions (including prediabetes, any kidney disease and hypertension which can all be quite mild from a patient-perspective) makes them able to identify more conditions. This is a contradiction. The authors also only compare the counts of included conditions compared to other definitions of multimorbidity but don’t explain the meaning between them and why their new definition might better serve their audience clinically. There is some mention of this around stomach issues being less common in Singapore. More of this depth of discussion is needed and should be brought together into a clear, convincing argument.

Response: Thank you for this comment. We would like to take this opportunity to clarify that addressing all the comments that the reviewer has kindly provided for our discussion section in this second round of revisions has, in our opinion, increased the depth and cohesiveness of our revised discussion section, in addition to having addressed the previous set of comments that were provided to us in the first round of revisions. With respect to the point on contradicting ourselves, we would like to clarify that multimorbidity definition is multi-dimensional and it is important to recognise that not all individual dimensions/parameters will point in the same direction of prevalence estimation (either increase or decrease). While the direction of prevalence estimation (increase or decrease) can be estimated and compared across different studies for individual dimensions/parameters (e.g., cut-off or number of included conditions), it is not plausible to exactly know the impact of a recommended definition on prevalence of multimorbidity when considering all parameters together. To further address this comment, we have made a distinction between the implications from epidemiological perspective versus clinical perspective in the revised manuscript as higher prevalence estimation does not necessarily mean more clinically meaningful definition of multimorbidity to avoid any confusion between the two, which may be interpreted as contradictory implications. We have added a new para in the revised discussion to incorporate above (please find copied below).

To address the point on why our cut-off of 3+ conditions is relatively better while also highlighting why our consensus-derived definition is more scientifically sound and accurate as compared to other local definitions, please refer to our response to comment 26 above. To address the point on only comparing counts of included conditions to other definitions of multimorbidity, we would first like to highlight that in the previously submitted manuscript, we have included discussion/comparison around type of conditions as well and not just limit to counts (comparing included chronic conditions with those reported in literature, comparing exclusion of acute conditions with the existing literature etc.). To further address the point of why our new definition might better serve the audience clinically, we have added new text expanding on clinically relevance of our recommended definition (in addition to previously stating that our definition will help identify patients with higher care needs who can be better managed in primary care setting). For further details, please refer to our response to comment 27 above. Hopefully, we have addressed the reviewer’s comment with our response.

“From an epidemiological perspective, while having a higher cut-off or threshold for measuring multimorbidity is associated with a lower prevalence, having a longer list of conditions is associated with a higher prevalence. Although looking at such individual dimensions for defining multimorbidity across different studies may present conflicting impact on prevalence estimation, it is important to understand that multimorbidity definition is multi-dimensional and hence, it is not plausible to exactly know the impact of a recommended definition on prevalence of multimorbidity when compared with others. Moreover, it is more meaningful to compare the clinical relevance of such definitions along with understanding the accuracy of developing such definitions. From a clinical perspective, our definition is more meaningful compared to local literature since with a higher cut-off, it would result in identification of patients with multimorbidity with higher care needs, who would benefit from the holistic management by a family physician in a primary care setting. It is not feasible to assess the accuracy of the local existing studies as none of them explained in detail the science behind developing their definition. In contrast, our recommended definition of multimorbidity, including the list of 23 conditions, was a result of consensus-voting by a group of Delphi panellists, who had both expertise in the field of multimorbidity and practical insights into managing patients with multiple chronic conditions in a primary care setting. Therefore, our multimorbidity definition and list of conditions are potentially more suited for defining and measuring multimorbidity within the Singapore primary care setting. In the absence of a universally recommended definition of multimorbidity with simultaneous presence of heterogenous ways of defining multimorbidity and lack of consensus, our chosen Delphi methodology is one of the most appropriate methods to gain consensus. Moreover, the conduct and reporting of our Delphi study was done in accordance with CREDES [37], which illustrates the robustness of our study and the validity of our findings. Throughout the study, we upheld the core tenets of the Delphi process, i.e., maintaining quasi-anonymity, practising iteration and controlled feedback, responses of the group being analysed in a statistical manner and compared with pre-defined consensus thresholds and sharing of results with Delphi panellists after each round. All of the above led to the final recommendation of a consensus-derived, context-based, clinically relevant definition of multimorbidity, which was rooted in sound methodology and well-supported by our study findings.”

Comment 33: Discussion

-The discussion is quite long and should be tightened on rewrite.

Response: Thank you for this comment. To address this comment, while re-writing the discussion in the revised manuscript (to address comment #24), we have tried our best to tighten the description as much as possible.

The change is reflected throughout the discussion section of the revised manuscript.

Comment 34: Conclusion

-the authors state: “The 593 consensus-derived definition of multimorbidity is the presence of three or more chronic 594 conditions from a finalised list of 23 chronic conditions” however they also included what they call Risk Factors and Symptoms, and do not state that in their definition.

Response: Noted, thank you for highlighting this. We have amended the text in the conclusion to indicate that the finalised list is a list of 23 conditions which is inclusive of diseases, risk factors and symptoms.

The change is reflected in the revised manuscript, page – 50, lines 879 – 880.

Comment 35: Tables

-see comment on Table 3 above

Response: Noted, we have addressed comment 13 above related to Table 3. Please refer to our response to comment 13 for more details.

Comment 36: Tables

-The authors claim Table 5 cannot fit onto 1 page but it clearly can fit onto 2 with some simple editing. Even though the journal allows multiple pages, I encourage the authors to take into consideration readability of the paper for their audience and take the reviewer’s suggestion to make this table more readable.

Response: Noted, we have amended the Table 5 in revised manuscript to fit it in 2 pages and address this comment.

**Reviewer 2:**

Comment 1: Thank you so much for the changes and further explanations made in accordance with the comments.

No further comments from my side.

Response: Thank you so much for taking the time to review our paper and providing us with positive and encouraging feedback. We are very grateful.
