# Supplementary material for: Defining and measuring multimorbidity in primary care in Singapore: Results of an online Delphi study
Source: PLoS One. 2022 Dec 1;17(12):e0278559. doi: 10.1371/journal.pone.0278559 (PMC9714819; doi:10.1371/journal.pone.0278559)
Supplement: Attachment — Submitted filename: Response to Reviewers.docx [file pone.0278559.s008.docx]

Prof (Dr) Gerald Koh, MD, PhD

Clinical Director and Head, Future Primary Care (FPC)

MOH Office for Healthcare Transformation

1 Maritime Square, #12-10, Harbourfront Centre

Singapore 099253

[Gerald_koh@moht.com.sg](mailto:Gerald_koh@moht.com.sg)

Hugh Cowley

Senior Editor, PLOS ONE

August 15, 2022

Re: Resubmission of manuscript *Defining and measuring multimorbidity in primary care in Singapore: results of an online Delphi study,* Manuscript ID PONE-D-21-28758R2

Dear Mr Cowley,

Greetings.

Thank you so much for seeing merit in our manuscript, entitled “Defining and measuring multimorbidity in primary care in Singapore: results of an online Delphi study,” and giving us the opportunity to revise it further. We really appreciate the feedback we have received.

We once again want to thank you and the reviewers for taking the time and effort to carefully read our work and provide us encouraging feedback, with constructive suggestions to work on. We believe that the manuscript is significantly improved after making the suggested amendments across the three rounds of revisions and we hope that it meets your expectations as well.

The revision has been developed in consultation with all co-authors, and each author has given approval to the final form of this revision. Changes made in the manuscript are marked using track changes in the “Revised Manuscript with Track Changes”. We have uploaded this track-changed copy along with a clean copy designated as “Manuscript for your kind perusal. We have no conflicts of interest to declare.

**Please find below the point-by-point response to all the comments by the Reviewers as required**.

Thank you for your kind consideration.

Sincerely,

Prof (Dr) Gerald Koh, MD, PhD

Clinical Director and Head, Future Primary Care (FPC), MOH Office for Healthcare Transformation

Professor, Saw Swee Hock School of Public Health, National University of Singapore

**POINT-BY-POINT RESPONSE TO COMMENTS BY REVIEWERS.**

Please take note that the page and line numbers mentioned in the responses below are referring to the revised version of the manuscript (marked copy with track-changes).

**Editor Comments.**

Comment: Please note the comments from Reviewer #1 below. The reviewer has noted in their report that they were not able to access some of the review files; I therefore contacted them with the missing documents, and they have now also been able to assess the changes you have made in full. As such, in addition to the comments in their report copied below, please also provide a response to the reviewer's follow-up comments:

Response: Thank you so much for taking the time to review our paper and providing us with this second round of comments/suggestions to improve our manuscript. We have tried to address the reviewer’s comments and made amendments as required. Hopefully, the revised manuscript will meet your standards.

**Reviewer 1:**

Comment 1: "I have reviewed the author’s response to reviewers. They have many changes successfully. However, for other recommended clarifications, they have added long explanations that are vague and do not get to the heart of the issue that I raised. Some concerns were not addressed or only partially addressed. More concise, terse changes would have benefitted the article.

Response: Thank you so much for giving us the opportunity to revise our manuscript and also acknowledging our efforts of revising our manuscript for past 2 rounds of reviewer comments. We have tried our best to provide adequate and relevant explanations to meet/address the comments kindly given by the reviewer in previous rounds. Additionally, we have tried our best to address any pending specific concerns/comments raised by the reviewer in this 3rd round of comments. Hopefully, by making the required revisions in this 3rd review round will address all pending comments.

Comment 2: While this is interesting work, and seems to have been done well, I still have trouble with the underlying premise that a single country, single practice setting definition of multimorbidity is needed or would be beneficial. I could see this doing harm to the clinical and research communities by causing division and increased heterogeneity in measures. The authors rationale is not clearly explained, despite my comment to them that it should be supported more clearly, and their rationale is at times contradictory."

Response: Thank you so much for this comment. As explained in the previous two rounds of revisions, we would like to take this opportunity to reiterate our intention was not to internationally benchmark and recommend a definition that is universally accepted, rather our study had a defined scope operationalising multimorbidity within our context, based on current existing literature and consensus voting by local experts from an implementation perspective. Furthermore, by engaging the stakeholders (i.e., the expert Delphi panellists) and having a comprehensive representation from the primary care sector of Singapore would hopefully advance the development of interventions to improve outcomes for patients with multimorbidity within Singapore. Since our study is based on existing literature and selection of relevant content by the Delphi methodology involving local experts, we are not generating a de novo definition of multimorbidity and hence our study is unlikely to add to the existing heterogeneity in the multimorbidity literature.

We apologise for limited clarity in our previous response to reviewer comment on why Singapore based, primary care specific definition of multimorbidity is needed. We would take this opportunity to further clarify the revisions included in the manuscript which justify this approach as follows: (1) prevalence of multimorbidity varies by care setting, (2) reliability of measures for multimorbidity used in one setting may not translate to another, (3) many studies have previously adopted a care setting specific approach, (4) we chose to focus on primary care setting from a management/practical/implementation relevance perspective, (5) it has been reported that “locally-generated research evidence” is more likely to be translated to practice as it is contextually more relevant and highly valued by policy makers and practitioners, and (6) Complementary to point (5) is the involvement of local stakeholders in the consensus building process, which will facilitate the acceptance of definition and following practical interventions subsequently. All of the above 6 points have been supported by cited literature in the revised manuscript.

We have amended this text to further clarify and make it more explicit to address the reviewer’s current comment. Also, we have added additional references to support the explanation that the prevalence of conditions vary by geographical regions and/or care settings, requiring a varying list of conditions for defining multimorbidity and our adoption of a region specific, care setting specific approach from a management perspective (as opposed to an epidemiological/international benchmarking perspective). Hopefully, we have adequately clarified and addressed this reviewer comment.

The change is reflected in the introduction section of the revised manuscript on page – 5-6 and lines 115 to 136

Comment 3: Reviewer #1: I appreciate the opportunity to review this paper again and appreciate the authors’ attempts to address review concerns. The paper is much clearer and more complete. The methods and results are now clear. The manuscript fills an important gap in the literature for Singapore primary care and perhaps beyond. However, from the authors efforts to incorporate the reviewer’s comments, the Introduction and Discussion are now much too long for a standard journal article. I was also unable to find the Response to Reviewers comments within the online reviewer platform.

Response: Thank you so much for acknowledging our efforts to revise our manuscript and appreciating the clarity and completeness of our revisions. We have revised the introduction and discussion section to make it more concise while trying our best to keep the content meaningful and coherent.

The change is reflected in the introduction and discussion section of the revised manuscript.

Comment 4: The Tables are much improved and add to the understanding of the methods and results.

Response: Thank you so much for this positive feedback.

Comment 5: Please revise the Introduction and Discussion to be shorter. The content is good but a terse re-write is needed.

Response: Thank you so much for this comment, we have revised the introduction and discussion section to make it shorter while trying our best to keep the content meaningful and coherent.

Comment 6: -The paper needs to be consistent about the multimorbidity measure being for Singapore with the potential to be used broadly. Often it come across that this is a general use measure and the study design does not support that.

Response: Thank you so much for this feedback. To address this comment, we have reviewed the manuscript and revised where appropriate to make it further explicit that we are proposing a multimorbidity measure for use in Singapore with the potential to be used broadly.

The change is reflected throughout the manuscript.

Comment 7: -Introduction: “While the World Health Organisation has defined multimorbidity as “being affected by two or more chronic health conditions”,[13] the European General Practice Research Network 53adopted a more comprehensive approach and defined multimorbidity as “any combination of 54chronic disease with at least one other disease (acute or chronic) or bio-psychosocial factor 55(associated or not) or somatic risk factor.” [14] Agency for Healthcare Research and Quality defines multiple chronic conditions as presence of “two or more chronic physical or mental health conditions.”” Please check the gramma of the first sentence and incorporate the 2nd sentence with the first.

Response: Thank you so much for this comment. To address this, we have revised the grammar of the 2 sentences and incorporated the 2nd sentence with the first.

The change is reflected in the revised manuscript, page – 3, lines 52 to 60.

Comment 8: -Introduction: The authors state that previous measure do not define what a chronic condition is and that is false. The authors themselves later explain how chronic conditions are defined by various groups.

Response: Thank you for highlighting this. To incorporate this comment, we have removed this line from the introduction of the revised manuscript.

Comment 9: Discussion: “Our study found the most commonly reported cut-off for defining multimorbidity in 477qualitative Delphi Round 1 was 3 or more conditions.” Would be better revised as “Our study found the most commonly recommended cut-off for defining multimorbidity in qualitative Delphi Round 1 was 3 or more conditions”

Response: Thank you so much for this suggestion, we have incorporated this in the revised manuscript.

Comment 10: Discussion: Your argument that pain cannot be mapped to ICD-10 codes is superficial. There are certainly chronic pain codes in ICD-10 (G89.4 for one) and attempts have been made to list all pain codes. It does remain a challenge and I encourage you to rewrite this section around the challenge rather than stating there are not pain codes in ICD-10

Response: Thank you so much for this comment. To address this, we have amended the indicated text under the discussion section of the revised manuscript.

The change is reflected in the revised manuscript, discussion section, page –36, lines 575 to 589.
